# Fixed or random? On the reliability of mixed‐effects models for a small number of levels in grouping variables

**DOI:** 10.1002/ece3.9062

**Published:** 2022-07-24

**Authors:** Johannes Oberpriller, Melina de Souza Leite, Maximilian Pichler

**Affiliations:** ^1^ Theoretical Ecology University of Regensburg Regensburg Germany; ^2^ Department of Ecology University of São Paulo São Paulo Brazil

**Keywords:** fixed effects, generalized linear models, hierarchical models, mixed‐effects models, multilevel models, random effects

## Abstract

Biological data are often intrinsically hierarchical (e.g., species from different genera, plants within different mountain regions), which made mixed‐effects models a common analysis tool in ecology and evolution because they can account for the non‐independence. Many questions around their practical applications are solved but one is still debated: Should we treat a grouping variable with a low number of levels as a random or fixed effect? In such situations, the variance estimate of the random effect can be imprecise, but it is unknown if this affects statistical power and type I error rates of the fixed effects of interest. Here, we analyzed the consequences of treating a grouping variable with 2–8 levels as fixed or random effect in correctly specified and alternative models (under‐ or overparametrized models). We calculated type I error rates and statistical power for all‐model specifications and quantified the influences of study design on these quantities. We found no influence of model choice on type I error rate and power on the population‐level effect (slope) for random intercept‐only models. However, with varying intercepts and slopes in the data‐generating process, using a random slope and intercept model, and switching to a fixed‐effects model, in case of a singular fit, avoids overconfidence in the results. Additionally, the number and difference between levels strongly influences power and type I error. We conclude that inferring the correct random‐effect structure is of great importance to obtain correct type I error rates. We encourage to start with a mixed‐effects model independent of the number of levels in the grouping variable and switch to a fixed‐effects model only in case of a singular fit. With these recommendations, we allow for more informative choices about study design and data analysis and make ecological inference with mixed‐effects models more robust for small number of levels.

## Graphical abstract

Many questions around the practical applications of mixed‐effects models are solved but one is still debated: Should we treat a grouping variable with a low number of levels as a random or fixed‐effect? Here, we analyzed the consequences of treating a grouping variable with 2‐8 levels as fixed‐ or random‐effect in correctly specified and alternative models (under‐ or overparametrized models). We found no influence of model choice on type I error rate and power on the population‐level effect (slope) for random intercept only models, but with varying intercepts and slopes in the data‐generating process, using a random slope and intercept model, and switching to a fixed‐effects model, in case of a singular fit, avoids overconfidence in the results.

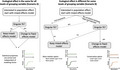

## 1 INTRODUCTION

Many biological data from experimental or observational studies have hierarchical grouping (or blocking, or clustering) structures that introduce dependencies among observations (Bolker et al., 2009; Harrison et al., 2018; McMahon & Diez, 2007; Zuur et al., 2009). A statistical analysis must account for these dependencies to ensure consistency of statistical properties (e.g., type I error rate) (Arnqvist, 2020), a task for which linear and generalized mixed‐effects models (LMMs or GLMMs) were designed (Chen & Dunson, 2003; Laird & Ware, 1982). Mixed‐effects models have replaced ANOVAs as the common tool for variance analysis (Boisgontier & Cheval, 2016; Bolker et al., 2009; Wainwright et al., 2007) because they allow simultaneous analysis of variance at different hierarchical levels (Boisgontier & Cheval, 2016; Krueger & Tian, 2004), handle unbalanced study designs better (Lindstrom & Bates, 1988; Littell, 2002; Pinheiro & Bates, 1995; Swallow & Monahan, 1984), and have better statistical properties for missing data (Baayen et al., 2008).

Mixed‐effects models have the ability to adapt to different data structures, but the flexibility (see Box 1; Wainwright et al., 2007) that comes with them also leads to discussions about their challenging application (Dixon, 2016; Nakagawa & Schielzeth, 2013). This includes data‐related properties such as the best way to handle overdispersion (Harrison, 2014, 2015), small sample sizes in the individual blocks (Gelman & Hill, 2007), technical aspects such as robustness to wrong distributional assumptions of the random effects (Schielzeth et al., 2020), and to questions about how to compare different mixed‐effects models (e.g., using *R* ^2^, Nakagawa & Schielzeth, 2013). Additionally, there are application‐oriented issues (Harrison et al., 2018; Meteyard & Davies, 2020) such as the question about the complexity of the random‐effect structure (Barr et al., 2013; but see Matuschek et al., 2017), the interpretation of random effects (e.g., Dixon, 2016), or when a grouping variable should be treated as random or fixed effect (Harrison et al., 2018).

A priori, modeling a grouping variable as fixed or random effect are for balanced study designs equally well suited for multilevel analysis (Kadane, 2020; Townsend et al., 2013). There are no strict rules, because the best strategy generally depends on the goal of the analysis (Gelman & Hill, 2007, see Box 2), however, for unbalanced designs there are some subtilities. For instance, random‐effect estimates incorporate between and within group information, whereas the corresponding fixed‐effects model (grouping variable is specified as a fixed effect) only within group information which leads to different weighting of the individual level estimates (not in balanced study designs) (McLean et al., 1991; Dixon, 2016; Shaver, 2019; but see Giesselmann & Schmidt‐Catran, 2020).This is important when one is interested in the actual‐level effects themselves (narrow‐sense inference analysis), but also when only interested in the population‐level effect (broad‐sense inference analysis), i.e., where the individual levels of the grouping variable are not of interest and one uses a non‐linear model. For this type of analysis, for a fixed‐effect model, we cannot simply build the weighted average over the individual levels to obtain the population‐level effect, because the non‐linearity does not commute with the expectation value.

The different inferential conclusions that result from fixed‐ and random‐effect modeling are due to the different assumptions underlying these two options (Millar & Anderson, 2004). Modeling a grouping variable as random effect implicitly assumes that the individual levels of the grouping variable are realizations of a common distribution, usually a normal distribution, for which the variance and the mean (the population‐level effect) need to be estimated (e.g., DerSimonian & Laird, 1986). As random effects are commonly parametrized so that the random‐effect has a zero mean, this assumption shrinks the estimates of each random‐effect level to zero. In contrast, treating a grouping variable as a fixed effect makes no distributional assumptions about the individual level estimates (i.e., treating the levels separately of each other and, thus, no between‐level information is used to estimate the level effects). The random‐effect model has fewer effective parameters than the fixed‐effects model because of the shrinkage (e.g., Gelman & Hill, 2007) which can lead in balanced designs to higher statistical power to detect significant population‐level effects at the cost of higher computational and numeric demand (Bolker et al., 2009), discussions on how to correctly calculate *p*‐values in unbalanced designs (Bolker et al., 2009; see Nugent & Kleinman, 2021) and a bias towards zero of the random‐effect estimates (Johnson et al., 2015).

So, if we are not interested in each individual‐level effect (broad‐sense inference), random‐effect modeling seems preferable over fixed‐effects modeling. It is, however, unclear if these advantages remain when the number of levels in the grouping variable is small (cf. also Harrison et al., 2018), because this might cause an imprecise and biased random‐effects variance estimate (Harrison et al., 2018), which then could influence the population‐level effect estimate of the mixed‐effects model (Hox et al., 2017).

The ecological literature suggests, as a rule of thumb, that an approximately precise estimate of the random‐effect variance requires at least five, sometimes eight, levels (Bolker, 2015; Harrison, 2015; Harrison et al., 2018). With four or fewer levels in the grouping variable, the preferred alternative is to include it as a fixed‐effect (Bolker, 2015; Bolker et al., 2009; Gelman & Hill, 2007). But this threshold seems to be arbitrarily chosen as it varies by discipline, e.g., 10–20 in psychology (McNeish & Stapleton, 2016), or 30–50 in sociology (Maas & Hox, 2005). To our knowledge, however, none of these values were based on a systematic analysis of how the modeling choice of the grouping variable affects statistical properties such as the type I error rate and power of the estimated population‐level effects (i.e., the weighted average slope or intercept over a grouping variable).

Here, we analyze a situation where an analyst wants to infer the population‐level effect and decided to use a mixed‐effects model but is confronted with a low number of levels in the grouping variable. For this scenario, we simulated an unbalanced study design on the height of a plant on a temperature gradient to compare empirical power and type I error with a varying number of levels (two to eight mountains). To represent the challenge of correctly specifying the model structure and the consequences if the structure is not correctly specified, we additionally tested mis‐specified models (overparametrized or underparametrized versions of the fixed and mixed‐effects models). To quantify the effect of these modeling choices on the population‐level effect, we compared: type I error rates and statistical power. Based on our results and in the context of broad‐sense inference, we give practical recommendations on when to include grouping variables as random effect or as fixed effect.

BOX 1 **Scenario of an ecological study design with grouping/blocking variables**

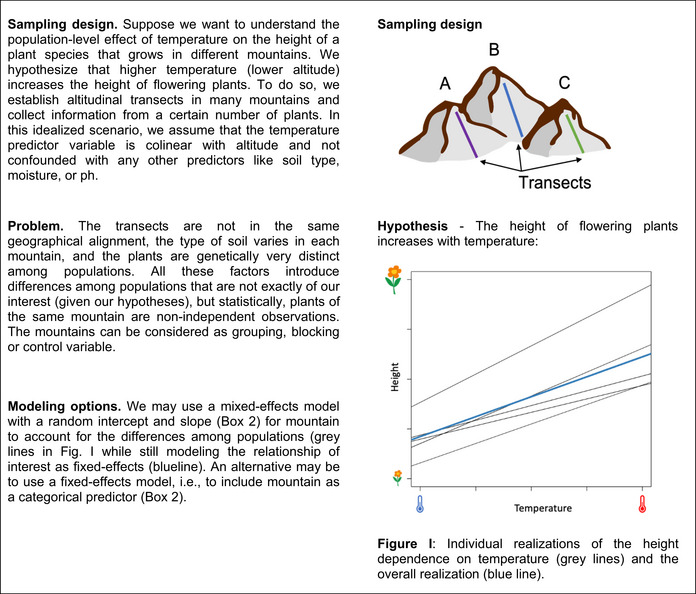

BOX 2 **Modeling a grouping variable as random or fixed‐effect**

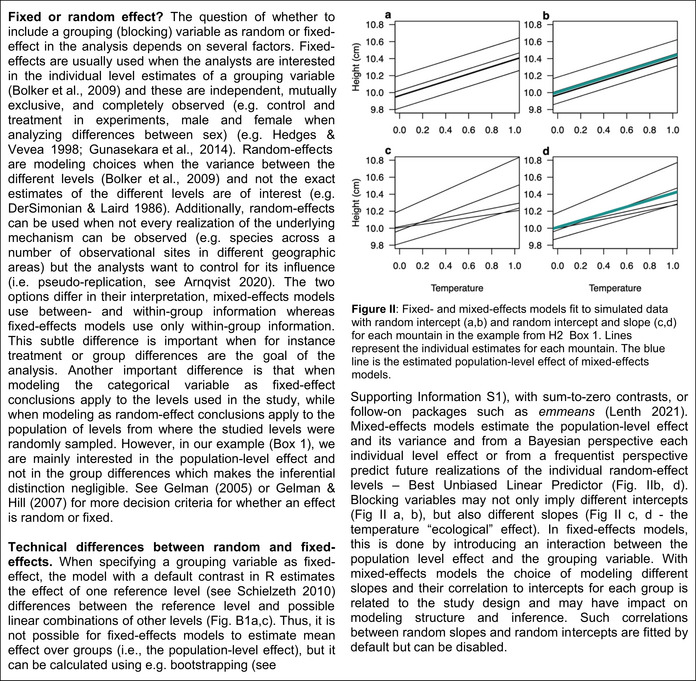

## 2 METHODS

### 2.1 Simulated example and scenarios of data and model complexity

To compare random‐ and fixed‐effects modeling of a grouping variable with small number of levels, we simulated data based on our hypothetical example from Box 1. We hypothesized, that higher temperatures increase the average height of plants. We simulated an unbalanced study design – a common scenario in ecology and evolution (Schielzeth et al., 2020) – with two to eight mountains and a varying number of plants for each mountain (expected range between 40–360 plants per mountain) while keeping the overall number of plants constant (on average 200 plants per mountain) along altitudinal transects. For each case, we simulated 5000 datasets.

#### 2.1.1 Scenario A ‐ random intercepts per mountain

In scenario A, we assumed mountains only differ in their intercepts (mean height), and the effect of temperature (slope) is the same for each mountain (constant slope over the levels of the grouping variable, Table 1, Equation M1). We tested two different mixed‐effects model structures: a correctly specified model which corresponds to the data‐generating process (Table 1, Equation M4) and an overparametrized model (Table 1, Equation M5) with an additional random slope for each mountain. Since in real studies the true underlying data‐generating process is unknown, it is useful to understand if an overparametrized model correctly estimates the variances of the random effects to zero and predicts all random slope levels to zero (or nearly zero) and, thus, approximate the data‐generating process (Table 1, Equation M1).

**TABLE 1:** Data‐generating and tested models for each scenario: Scenario A random intercept for each mountain, and B random intercept and slope for each mountain. For the fixed‐effects models, we used R syntax for model formula in *lm()* function, and for the mixed‐effects models we used syntax from *lmer* functions from *lme4*. The response variable is height of flowering plants (H1, Box 1) and T is the temperature effect

	Scenario A		Scenario B		Description
	Random intercept only		Random intercept and slope		
Data‐ generating model	(M1)	Height ~ T + (1|mountain)	(M6)	Height ~ T + (1|mountain) + (0 + T|mountain)	Effect of intercept (and slope in B) vary across mountains
Tested models					
Fixed‐effects models	(M2)	Height ~ T	(M7)	Height ~ T	Temperature only main effect – underparametrized model
	(M3)	Height ~ 0 + T + mountain	(M8)	Height ~ 0 + mountain + T:mountain	Main effects of temperature and mountain (and interaction in B) – slightly more complex model
Mixed‐effects models			(M9)	Height ~ T + (1|mountain)	Temperature and mountain both vary – underparametrized models
	(M4)	Height ~ T + (1|mountain)	(M10)	Height ~ T + (1|mountain) + (0 + T|mountain)	Effect of intercept (and uncorrelated slope temperature in B) vary across mountains – correctly specified models
	(M5)	Height ~ T + (1|mountain) + (0 + T|mountain)	(M11)	Height ~ T + (T|mountain)	Effect of intercept and slope temperature (correlated effects in B) across mountains – overparametrized models

As fixed‐effect alternatives, we tested the correctly specified model with mountain as fixed intercept together with temperature as slope (Table1, Equation M3), and an underparametrized model omitting mountain at all (Table 1, Equation M2). This last model corresponds to a mixed‐effects model that estimates the variances of the random effect to be zero and thus predicts the random effects to be zero.

#### 2.1.2 Scenario B ‐ random intercepts and random slopes per mountain

In scenario B, we assumed the data‐generating process contained a random intercept and a random slope (without correlation among the random slopes and intercepts) for each mountain (Table 1, Equation M6). Here, the population‐level effect (temperature) differs among levels of the grouping variable (mountain). We tested three different mixed‐effects model structures: a correctly specified model corresponding to the data‐generating process (Table 1, Equation M 10), an overparametrized model containing an extra term for the correlation of the random intercept and random slope (Table 1, Equation M 11), and an underparametrized model with only a random intercept for each mountain (Table 1, Equation M 9). We used the underparametrized model to test the effect of not accounting for important contributions to the data‐generating process. Note, however, only in case of balanced designs and linear models the population‐level effect estimate from the underparametrized model is consistent with the full model, because of different weighting schemes (for unbalanced designs), and the fact that the expected value of a non‐linear transformation of estimates is not the same as the non‐linear transformation of the expected value of these estimates.

As fixed‐effect alternatives, we tested the correctly specified model with the main effects of temperature, mountain, and their interaction (Table 1, Equation M 8), and the underparametrized model without mountain as predictor (Table 1, Equation M 7). We tested the last model because mixed‐effects models that estimate zero variance for both random effects are virtually the same as fixed‐effects models that omit the grouping variable.

### 2.2 Model fitting

We fitted linear mixed‐effects models to our simulated data with the *lme4* R package (Bates et al., 2015) together with the *lmerTest* (Kuznetsova et al., 2017) package, which uses the Kenward‐Rogers approximation to get the *p*‐values of the fixed‐effects. For fixed‐effects models, we used the *lm()* function of the R *stats* package (Version 4.1, R Core Team, 2021). For fixed‐effects models in scenario A, we extracted *p*‐values from the *summary()* function and, for scenario B, we used the fitted variance–covariance matrix and the individual‐level effects to bootstrap the population‐level effect and its standard error (see Supporting Information S1).

Obtaining *p*‐values for mixed‐effects models is intensively discussed in the statistical community and they are only exact for simple designs and balanced data (Kuznetsova et al., 2017). One reason is that in order to calculate *p*‐values in mixed‐effects models, denominator degrees of freedom must be calculated, which generally can only be approximated (Kuznetsova et al., 2017). For best practice in which situations one should use which approximation see (Bolker et al., 2009; see also Nugent & Kleinman, 2021). The *lmerTest* package uses the Satterthwaite method to approximate the degree of freedoms of the fixed effects in the linear mixed‐effect model.

We used the restricted maximum likelihood estimator (REML) (for a comparison of REML and maximum likelihood estimator [MLE] see Appendix and Supporting Information S1). All results of mixed‐effects models presented in scenarios A and B are for the datasets without singular fits (see Section on *Variances of random‐effects and singular fits*). Technically, singular fits occur when at least one of the variances (diagonal elements) in the Cholesky decomposition of the variance–covariance matrix are exactly zero, or correlations between different random effects are estimated close to −1 or 1.

We repeated the analysis for the *glmmTMB* R‐package because it uses a different implementation to estimate mixed‐effect models (see Appendix for methods and results).

### 2.3 Statistical properties and simulation setup

We used type I error rate and statistical power of the population‐level effects (average height and temperature) to compare the modeling options. For example, type I error rate for the temperature (slope) is the probability to identify a temperature effect as statistically significant although the effect is zero. Statistical power in this case is the probability to detect the temperature effect as significant if the effect is truly greater than zero. For a correctly calibrated statistical test, the type I error is expected to be equal to the alpha‐level (in our case 5%).

To investigate type I error rates of the models on the intercept (average height) and average slope (temperature effect), we simulated data with no effects, i.e., the effects of temperature and mountain on height is zero. To additionally investigate statistical power, we simulated an example with a weak effect which corresponds to an average increase in size per unit step of the standardized temperature (linear scale) of 0.4 cm.

For scenarios A and B, the individual effects for each mountain were drawn from a normal distribution with variance of 0.01 and 0.25 around the average effects: 0.4 cm average height (intercept), and 0.4 cm average increase in size with temperature (slope). We chose to run and compare simulations with these two values for the variance of the random effects to understand better how a larger or smaller variance may interfere in type I and power.

### 2.4 Variances of random effects and singular fits

To understand how the number of levels affected random‐effects variance estimates, we compared the variance estimates for random intercepts and slopes from the correctly specified mixed‐effects model in scenario B (Table 1, Equation M10). We also compared optimization routines (REML and MLE) in terms of estimating zero variances (singular fits, see below) (see Supporting Information S1). For bounded optimizations, which most R packages apply for the variance, it has been shown that the null distribution of a random effect's variance is a combination of a point mass at zero and a chi‐squared distribution (Stram & Lee, 1994). For the sampling distribution with a true variance unequal to zero there are no proofs, but one would expect a similar distribution.

While singular fits do not signal a convergence issue, the consensus is that the results of such models are not reliable. However, we decided to use non‐singular fits and additionally non‐singular and singular fits combined for calculating power and type I error for the mixed‐effects models, and to infer the effect of singular fits on the averaged statistical properties. We classified a dataset as singular or non‐singular if the mixed‐effects model ran in *lme4* reported a singular fit warning message. For fixed‐effects models, we used estimates from non‐singular and singular datasets combined.

Using only non‐singular fits for calculating power and type I error impacts these statistical properties (e.g., type I error) because they are conditional on this selection and thus likely not to be at the nominal level (e.g., 5% for type I error rate). However, as our main intention is to report the type I error rates from the point of the analyst who may adjust the model structure to dispose of the singular fit, our reported rates represent empirical type I error rates.

### 2.5 Quantifying the influences of study design on power and type I error

Power and type I error of the population‐level effect may depend not only on the number of levels (mountains) but also on the random‐effect variance, the overall number of observations and the balance of observations among levels. To further quantify the impact of these study design factors on statistical power and type I error rate of the population‐level effect, we additionally ran 1,000 iterations (each with 1,000 non‐singular model fits) with the data‐generating process from scenario B for our ecological example. Thereby, we sampled the number of mountains from 2 to 20 with equal probability for each number, the random‐effects variances from 10^−4^ to 4, the overall number of observations from 10 to 500 times the number of mountains. Additionally, to create different degrees of unbalance in data, we sampled for each mountain the average share of total observations from 0.1 to 0.9, which corresponds to at least 3 observations per mountain. We used the difference between the largest and the lowest proportion as proxy for the degree of unbalance.

For the so‐generated data, we fitted the correctly specified linear mixed‐effects and fixed‐effects models from scenario B (Table 1, Equations M8 and M10) and calculated type I error rate and statistical power of the population‐level effect. We then fitted a quantile regression using the *qgam* R‐package (Fasiolo et al., 2020), with the statistical property (power and type I error rate) as response and variance, number of levels, total number of observations and the unbalance proxy as splines. We used a quantile regression with splines as we expect a non‐linear relationship.

## 3 RESULTS

### 3.1 Scenario A ‐ random intercepts per mountain

**FIGURE 1:**
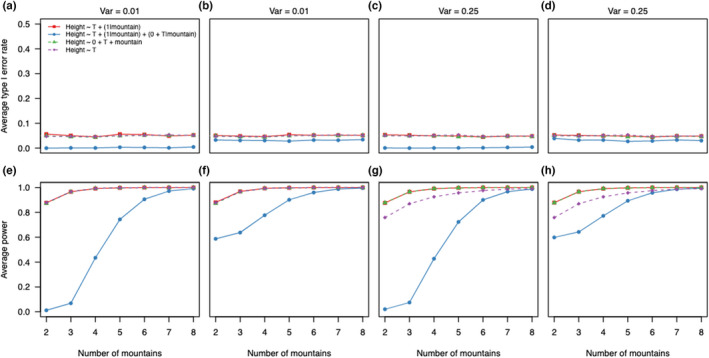
Average type I error rates and average power for linear fixed‐ and mixed‐effects models fitted to simulated data with 2–8 mountains (random intercept for each mountain ‐ Scenario A). For each scenario, 5,000 simulations and models were tested; (a, b, e, f) show results for simulated data with a variance of 0.01 in the random effects; (c, d, g, h) show results for simulated data with a variance of 0.25 in the random effects; (a, c, e, g) show results for mixed‐effects models only from datasets in which mixed‐effects models converged without presenting singular fit problems and (b, d, f, h) results for mixed‐effects models for all datasets. Results for fixed‐effects (a‐h) model are from all datasets. (a‐d) the dotted line represents the 5% alpha level

When the effect of the temperature predictor was the same among mountains, irrespectively of the number of levels (mountains), all models except for the overparametrized model (random intercept and slope) showed an average type I error rate of 5% (Figure 1a–d). Average power increased (Figure 1e‐h) with the number of mountains from 90% (2 mountains) to 100% (5–8 mountains). Note that the model omitting the grouping variable presented similar properties as the other models for small variances in the random effect. However, when increasing the variance of the random intercept in the simulation, the model omitting the grouping variable showed lower power (Figure 1g,h).

For the overparametrized model, we found, on average, a lower type I error rate of less than 5% (Figure 1a‐d), and lower average statistical power to detect the temperature effect for a small number of mountains (Figure 1e‐h). When combining singular and non‐singular fits, the overparametrized model had more average power compared to only non‐singular fits and an average type I error closer to the nominal level (Figure 1).

The results for the intercept for the different models (see Figure S9) are similar to the results for the slope in scenario B (see below).

### 3.2 Scenario B ‐ random intercepts and slopes per mountain

**FIGURE 2:**
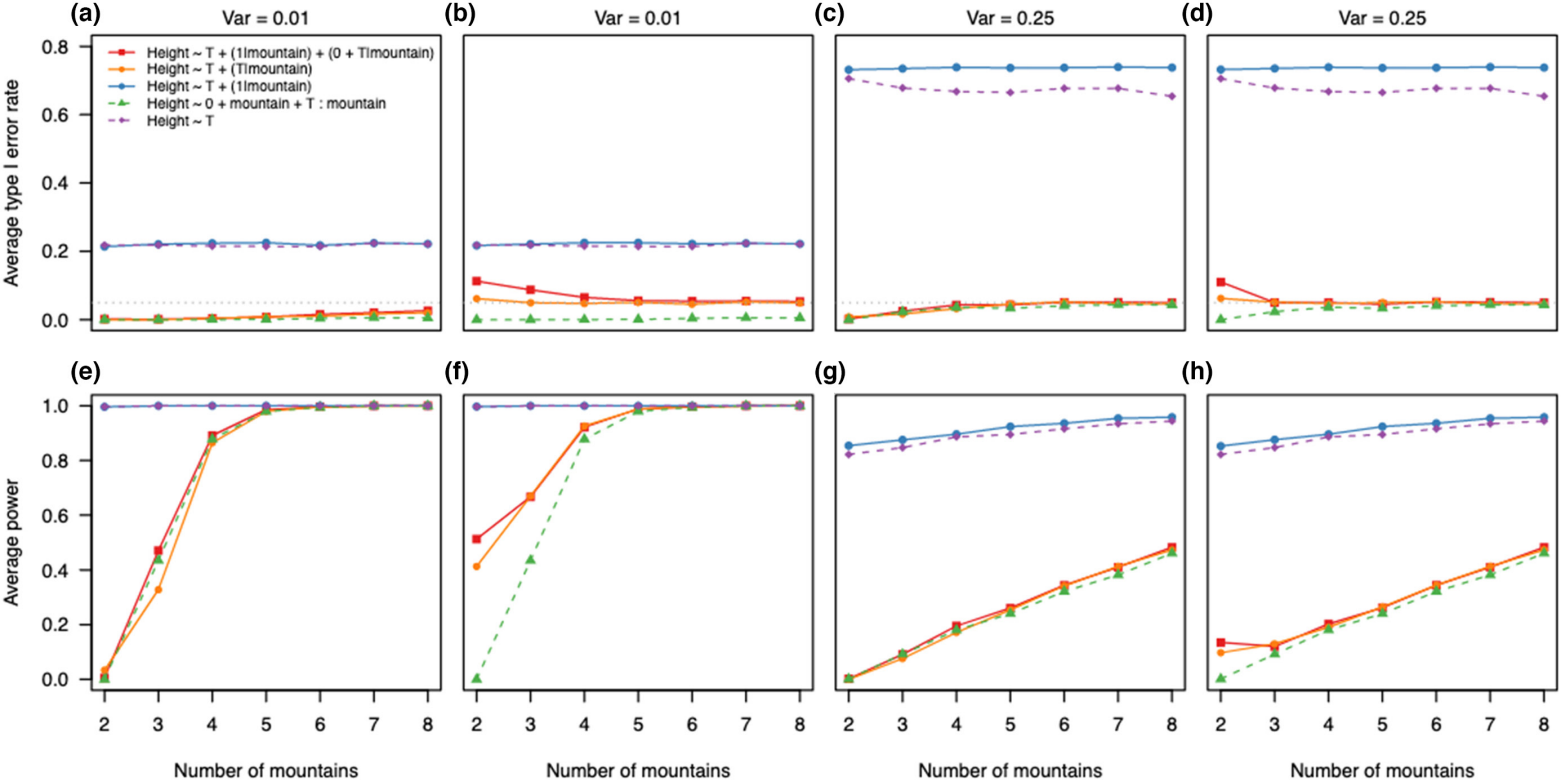
Average type I error rates and average power for linear (mixed‐effect) models fitted to simulated data with 2–8 mountains for scenario B (random intercept and random slope for each mountain range). For each scenario, 5,000 simulations and models were tested; (a, b, e, f) show results for simulated data with a variance of 0.01 in the random effects; (c, d, g, h) show results for simulated data with a variance of 0.25 in the random effects; (a, c, e, g) show results for mixed‐effects models only from datasets in which mixed‐effects models converged without presenting singular fit problems and (b, d, f, h) results for mixed‐effects models for all datasets. Results for fixed‐effects (a‐h) model are from all datasets. In (a‐d) the dotted line represents the 5% alpha level

In scenario B, where the effect of the temperature differed among levels, the modeling decision influenced the average power and average type I error (Figure 2). We found that average type I error rate of the correctly specified mixed‐effects model (Table 1, Equation M10) slightly increased (Figure 2a) with the number of levels towards the nominal value (0.05) (Figure 2a). The increase was stronger for larger variances (0.25) in the random effects (Figure 2c). With singular fits, the mixed‐effects models showed a higher average type I error rate than the nominal level for lower number of mountains (Figure 2b, d). With a higher variance in the random effects, the average type I error rate was only increased for two levels (Figure 2d). The overparametrized model with correlated random intercept and random slope (Table 1, Equation M11) presented similar properties, but with decreased average power (Figure 2e‐h).

For the correctly specified fixed‐effects model, average type I error (**≈** 2%) stayed constant with the number of levels (Figure 2c) and a low variance in the random effects but increased stronger to the nominal level with a higher variance (Figure 2d). Average power increased with the number of mountains (Figure 2e‐h). The mixed‐effects model showed higher average power than the fixed‐effects model irrespective of the number of mountains (Figure 2e‐h).

The underparametrized model without the grouping variable had a higher average type I error rate (0.2) and higher average power than the other models (Figure 2e‐h). With a higher variance, the average type I error rate was even higher (0.8; Figure 2c, d).

### 3.3 Variance estimates of random effects and singular fits

**FIGURE 3:**
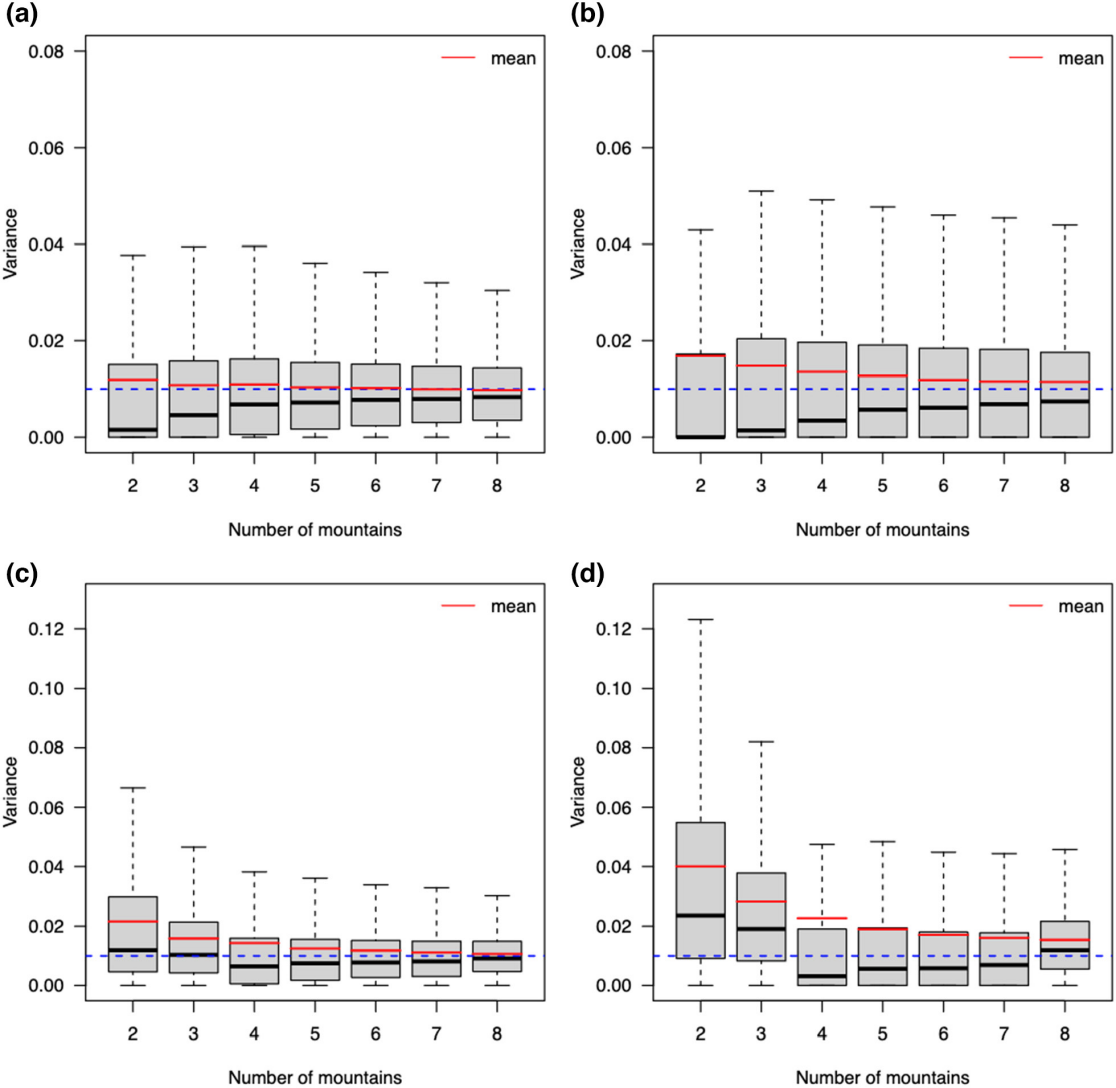
Variance estimates of random intercepts (a, c) and random slopes (b, d) for linear mixed‐effects models (LMM, Table 1. Equation M10) in Scenario B, fitted with *lme4* using REML to simulated data with 2–8 mountains. Figures (a) and (b) show the results for all models (singular and non‐singular fits) and figures (c) and (d) show the results for only non‐singular fits. For each scenario, 5,000 simulations and models were tested. The blue dotted lines represent the true variance used in the simulation (0.01), and the red lines the average variance estimates

We found, for the models (singular and non‐singular fit results combined) in Scenario B (random intercept and slope) that random‐effects' variance estimates of the correctly specified model (Table 1, Equation M10) approximately distributed as a chi‐squared distribution around the correct value (0.01) and a point mass at zero (Figure 3a,b median is near to zero). The point mass at zero decreased in height with increasing number of levels, i.e., less models estimated a variance of zero with an increasing number of mountains (Figure 3a,b, see also Table S1). There was smaller bias for the random intercept variance estimates than for the random slope variance estimates, which were still biased for eight levels. When looking at models without singular fits, the variance estimates were chi‐squared distributed (Figure 3c,d). The bias towards larger values was stronger compared to estimates with singular fits, especially for the random slope estimates (Figure 3d).

By comparing the fitting algorithms, we found that using MLE led to more zero‐variance estimates, i.e., singular fits, (Figure S3, S4) than REML. Additionally, using MLE, non‐singular variance estimates were strongly biased (Figure S3, S4), but the bias decreases with increasing number of levels. As expected, for both optimization routines, increasing the number of levels reduced the number of singular fits (Table S1).

**FIGURE 4:**
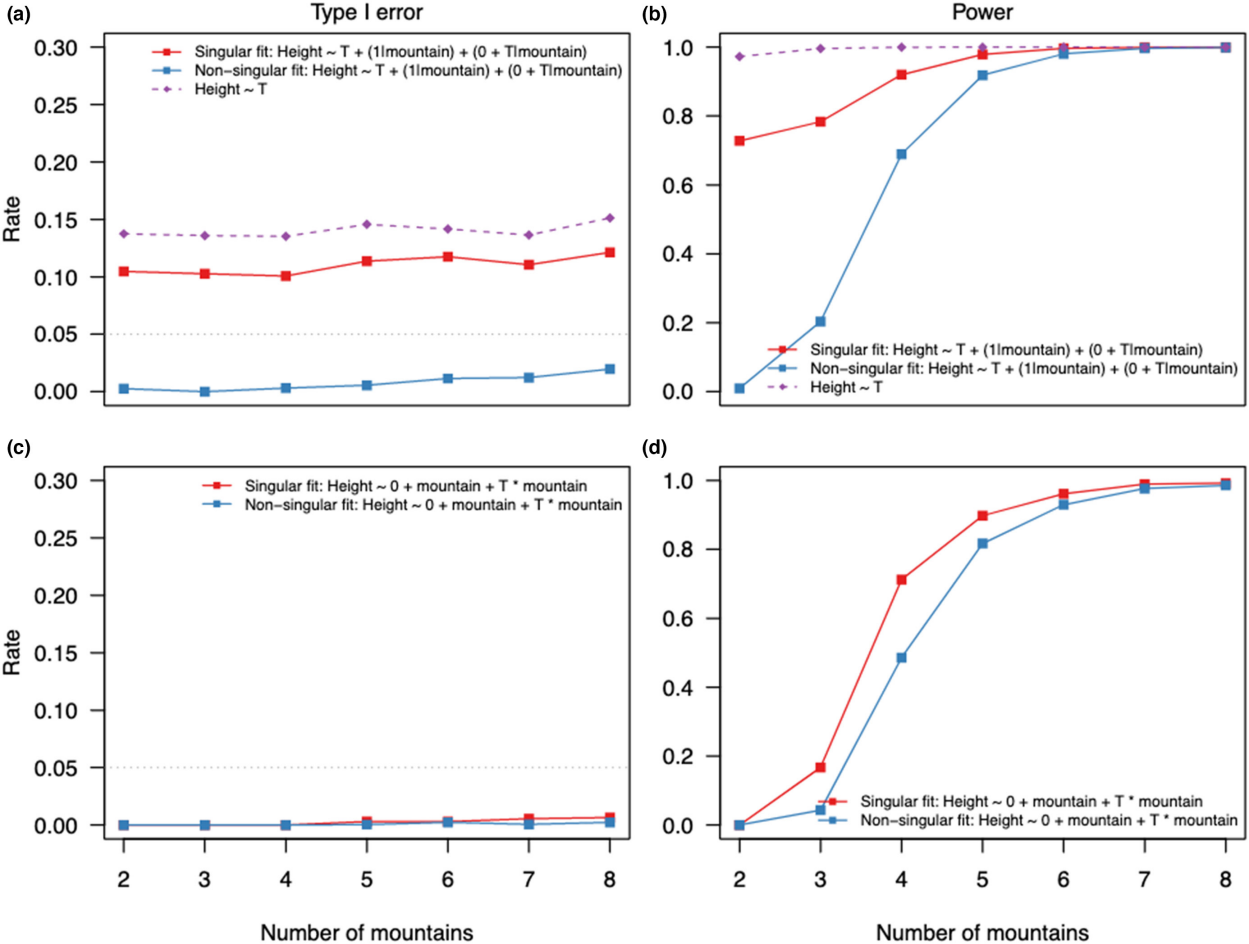
Type I error rate and power of the correctly specified linear fixed and mixed‐effects models in scenario B. We separated the datasets based on if when fitted they presented a singular fit (red lines) or non‐singular fit (blue lines) warning. Figure (a) and (b) are results for the linear mixed‐effects models, and (c) and (d) for the linear fixed‐effects models. For comparisons, we show also results for the fixed‐effects model that omits the grouping variable (mountain)

We found that singular fits led to different type I error rates and statistical power (Figure 4) in mixed‐ and fixed‐effects models. For singular fits, the type I error rate of the correctly specified mixed‐effects model was constant around 10% (like the model omitting the grouping variable), while with non‐singular fits it was 1% for two levels and increased towards 3% with eight levels (Figure 4a). In comparison, the fixed‐effects model had similar type I error rates (no distinction between singular and non‐singular fits because fixed‐effects models do not estimate the variance of the individual level estimates), both increasing from 0% (two levels) towards 1% (eight levels) (Figure 4c).

We also found differences in power for the mixed‐effects models between singular and non‐singular fits (Figure 4b, d). The power of the mixed‐effects model with correct structure was higher for singular than non‐singular fits especially for a low number of mountains (Figure 4b).

### 3.4 Quantifying the influences of study design on power and type I error

**FIGURE 5:**
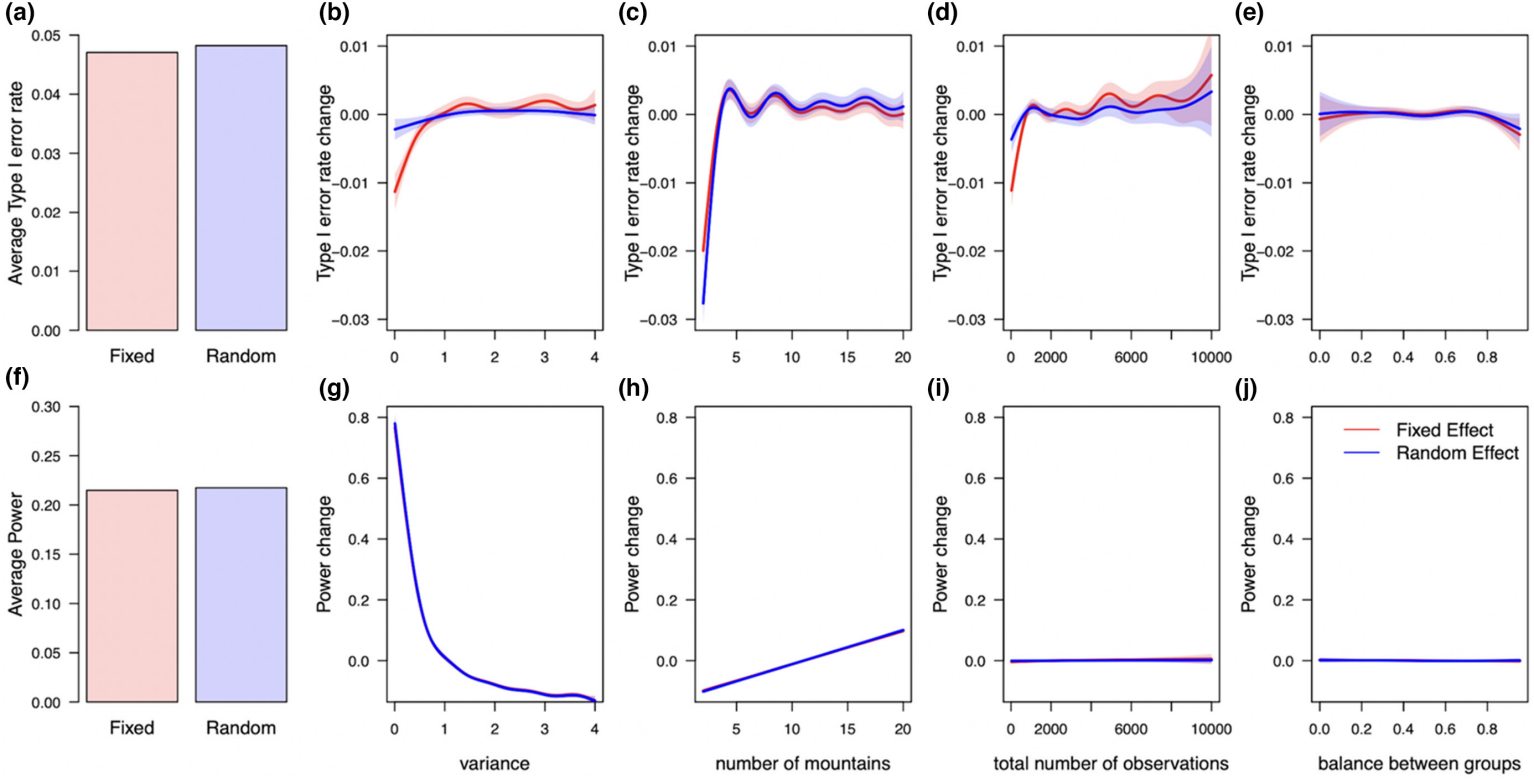
Comparing the influence of study design factors on the type I error rate (b ‐ e) and power (g ‐ j) of linear mixed‐ (blue lines) and fixed‐effects models (red lines) with their respective average values (a, f). We found that the variance of the random‐effects and the number of levels (*number of mountains*) are the most important values to get correct type I error. For this analysis, we used the plant height example for Scenario B (random intercept and random slope). Results for mixed‐effects models are only from datasets in which mixed‐effects models converged without presenting singular fit problems, while results for fixed‐effects model are from all datasets

We found that the average type I error of mixed‐effects models is slightly closer to the nominal value than its fixed‐effect counterpart (Figure 5a). Additionally, we found that the number of levels most strongly influences the type I error rate for mixed‐ as well as fixed‐effects model (Figure 5c). With five or more levels, however, the influence of the number of levels becomes negligible. Differences between the mixed‐ and fixed‐effects models arose for the variance and the total number of observations. Here, the mixed‐effects model was less influenced by a small random‐effects variance and a low number of total observations than the fixed‐effects model (Figure 5b,d). Balance, following our definition, (see Methods) did not influence the population‐level effect in both models (Figure 5e).

For power, we found no difference between a fixed‐ and mixed‐effects model (Figure 5f‐j). For both models, an increase in variance decreased the power, while increasing the number of levels increased the power (Figure 5g,i). The total number of observations and the balance between groups had less influence (Figure 5h,j).

## 4 DISCUSSION

Ecological data collections or experiments produce data with grouping structures, and mixed‐effects models can account for these dependencies. The main questions we explored in this article were: “should analysts stick to the mixed‐effects model or fall back to a fixed‐effects model, when the grouping variable has few levels?”, and “how does this decision influence statistical power and type I error rate of the population‐level effect?” Here, we showed with simulations that mixed‐effects models with a small number of levels in the grouping variable are technically robust (Figure 2), and that the decision between random and fixed effect matters most when the effect size of the ecological predictor variable differs among levels (Figure 2).

When the effect of the ecological predictor is the same for each level of the grouping variable (scenario A, random intercept model), almost all models presented the same average power and average type I error (see also Gomes, 2021) (Figure 1a‐d). The only exception was the overparametrized model that presented too low average type I errors and lower average power (Figure 1). We speculate that the model was unable to correctly predict the additional random effects to zero. Notably, for scenario A, the underparametrized model omitting the grouping variable presented correct average type I error rate (Figure 1a‐d). However, this is illusive because average power decreased with increasing effect sizes of the random effects (Figure 1g, h). This confirms that the grouping variable needs to be included to correctly partition the variance among the different predictors (Bell et al., 2019; Gelman, 2005; Gelman & Hill, 2007). Also, including the grouping variable is mandatory if one is interested in the average intercept, otherwise it would cause inflated average type I error rates (see Figure S1; see the following section).

When the effect size of the ecological predictor differs for each level of the grouping variable (scenario B; random intercept, and random slope model), the average type I error and power were influenced by both model choice and the presence of singular fit warnings. The mixed‐effects models had a better average type I error than the fixed‐effects models, especially for a larger number of mountains (Figure 2). Power was comparable between mixed‐ and fixed‐effects models. But with non‐singular and singular fits combined, the mixed‐effects model had higher type I error rates and power than the fixed‐effects models. In both cases, the mixed‐effects models showed good type I error rates (about more or less than 5%) for a small number of levels.

Overparametrized mixed‐effects models presented in both scenarios slightly lower average type I error and average power compared to the correctly parameterized mixed‐effects model (Figures 1 and 2). This trade‐off between type I error and power is in line with Matuschek et al. (2017) for different model complexities. Overall, the overparametrized models are more conservative but have less power than the simplified models. We think these more conservative estimates are preferable over anti‐conservative estimates, because some analysists tend to try a variety of analyses and only report significant ones (Simmons et al., 2011), and more conservative average type I error counteract this procedure.

However, dropping the correlation structure between random effects should be carefully considered. It is possible that the type I error rate increases when no correlation in the model is assumed although there is one in the data‐generating process. Group‐mean centering of the population‐level effect may mitigate the requirement of assuming a correlation, but it also changes the interpretation of the model because the individual levels are not referenced to the population‐level effect anymore (they are now independent).

In scenario B, underparametrized models exhibited inflated type I errors (in line with Schielzeth & Forstmeier, 2009; Barr et al., 2013; Bell et al., 2019) but very high average power (Figure 2). We speculate that additional variance coming from the difference between levels in the grouping variable, which is not accounted, is attributed to the population‐level effect and causes overconfident estimates.

### 4.1 Variances of random effects and singular fits

The rate of singular fits was very high for the small number of levels (Figure 3; Table S1). In our simulations, singular fits corresponded to zero variance estimates of the random effects. The resulting distribution of variance estimates consisted of a right skewed chi‐squared distribution and a point mass at zero (many zeros corresponding to the singular fits) as expected (see Stram & Lee, 1994). The variance estimates were biased and imprecise with a small number of levels, but the bias decreased with the number of levels towards zero (McNeish, 2017). Removing the singular fits led to even more bias in the variance estimates (Figure 3c,d).

The biased variance estimates are caused by ensuring positive variances in the optimization routines (Bates et al., 2015; Brooks et al., 2017). In case of a singular fit, the correctly specified mixed‐effects model had similar power and type I error as a fixed‐effects model dropping the grouping variable (Figure 4): no difference between the levels, which corresponds to a fixed‐effects model without the grouping variable. However, the models still differed in their number of parameters (and degrees of freedom) which might explain the slight differences in power and type I error (Figure 4).When switching to fixed‐effects models for singular fits in the random effect, the type I error rate and power were similar to the random‐effect model with non‐singular fits (Figure 4).

### 4.2 Connection to study design

Earlier studies reported mixed recommendations about important study design factors. While some studies only stressed the importance of the total number of observations (Martin et al., 2011; Pol, 2012), we found, in accordance with Aarts et al. *(* 2014), that the number of levels and the variance between levels have a strong influence on type I error rates and power. Due to our simulation design, which automatically increases the number of observations when increasing the number of levels, we however, cannot perfectly separate the effects of number of observations and levels from each other.

The influence of the variance on power and type I error is mixed. On the one hand, increasing the variance had a positive effect on the type I error for both models but the fixed‐effects model was more strongly affected (Figure 5). The different distributional assumptions might explain this different behavior: the mixed‐effects model assumes the levels to be normally distributed and estimates the variance of the levels’ flexibly, whereas the fixed‐effects model makes no distributional assumptions. We speculate that the mixed‐effects model benefits from this informative distribution assumption in this edge case with less than five levels. On the other hand, increasing the variance over a certain value (Figure 5g) decreased the power of both models because more variance is explained by the difference between levels, and this increases the uncertainty of the slope effect estimate.

Given the strong influence of the number of mountains on type I error rates, we encourage to design a study with at least eight levels because with more than eight levels, the type I error rate was approximately not affected by the number of levels (Figure 5c). In our scenarios, the influence of the unbalanced number of observations between levels was small (Figure 5) confirming the robustness of mixed‐effects to unbalanced data (Pinheiro & Bates, 1995; Schielzeth et al., 2020; Swallow & Monahan, 1984). However, if possible one should try to balance the groups because despite the robustness of mixed‐effect models to an unbalanced design, it impacts the interpretation of the random effects and balanced studies create the least problems regarding the model option (Dixon, 2016). Moreover, the impact of study design on type I error and power stresses the importance of pre‐experiments and power analyses (e.g., Brysbaert & Stevens, 2018; Green & MacLeod, 2016; Johnson et al., 2015) to maximize the meaningfulness and efficiency of a study.

### 4.3 Practical suggestion

Before giving practical advice, we must recall the exact situation in which this manuscript acts. We assume that an analyst is interested in a population‐level effect, and that they have already decided to use a mixed‐effects model (broad‐sense analysis, not interested in the individual levels effects), but faces a small number of levels, so that our recommendations only apply to such situations.

In this situation, the variance estimates of the random effects stabilizes in a reasonable manner with at least five levels in a grouping variable (Figure 2). With less than five levels, variance estimates are biased to zero (Figure 3) though without an effect on the observed average type I error rates of the population‐level effect (Figures 1, 2). We rather found that the question of how to deal with a singular fit in the mixed‐effects model is more crucial than the actual number of levels. If there is a singular fit warning, switching to the fixed‐effects model leads to more conservative average type I error rates (Figure 2). Acknowledging that most singular fits occur with a small number of levels (Table S1), this might also explain the common rule of thumb to not fit a grouping variable as random effect if it has fewer than five levels (Bolker, 2015; Bolker et al., 2009; Gelman & Hill, 2007).

**FIGURE 6:**
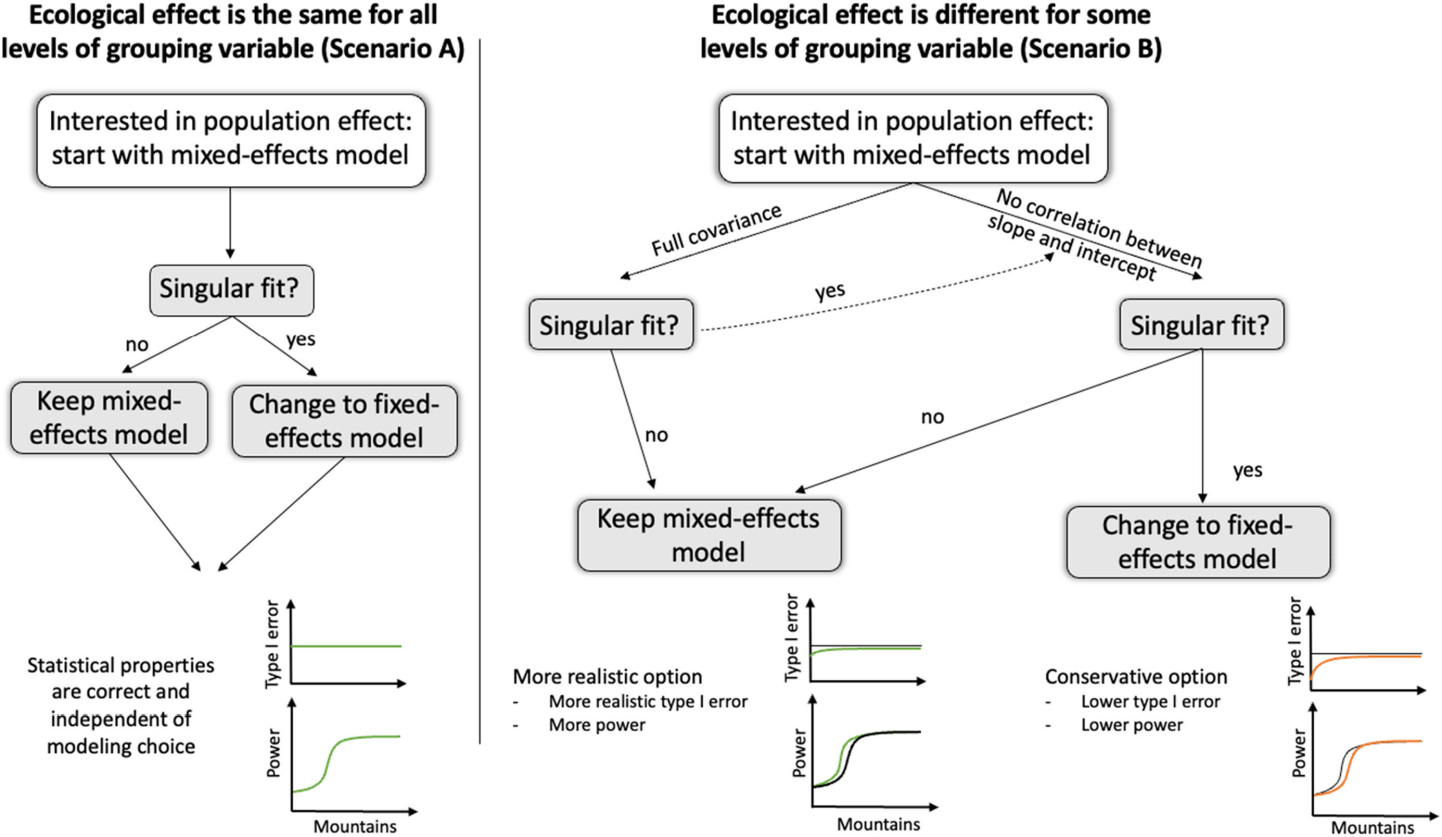
Consequences and recommendations for mixed‐effects models with a small number of levels in the random effect. When the ecological effect (population‐level effect) does not differ between different levels of the grouping variable (left side) all modeling options, which include the grouping variable, lead to the same results, and thus, only a singular fit requires a change to a fixed‐effects model. If the ecological effect (population‐level effect) differs among levels (middle to right side), starting with the mixed‐effects model and only changing to the fixed‐effects model in case of a singular fit is recommended

Our recommendations are summarized in Figure 6. We recommend starting with the mixed‐effects model, regardless of the number of levels, and switching to a fixed‐effects model only in case of a singular fit warning. How to deal with singular fits is a topic of ongoing discussion. While Barr et al. (2013) states to start with the maximum model and simplify the model in case of convergence issues and singular fits, Matuschek et al., 2017 suggests to think a priori about using simpler models because of higher power in return of increased type I error rate. However, we disagree with the view of (Matuschek et al., 2017) that trading a small increase in type I error rate for higher power is favorable, even though it could still be an interesting solution with the often‐small number of observations in ecological studies, when the increase in power prevails upon the increase in type I error rate. We follow the position of Barr et al. (2013), and thus recommend starting with correlated random slope and intercept, when the population‐level effect differs among levels. If obtaining a singular fit, switch to uncorrelated random‐effects (following Matuschek et al., 2017), and in case of another singular fit, switch to a fixed‐effects model.

Our recommendations assume that the random effect structure (e.g., random slope or not) is known a priori, which is often difficult in practice. Although model selection is theoretically possible for random effects (e.g., simulated (restricted) LRTs (Wiencierz et al., 2011) or by residual checks (as facilitated by Hartig, 2019), the frequentist point of view recommends sticking closely to the a priori‐derived hypothesis, otherwise the risks such as they arise from multiple testing increase. Moreover, if the grouping variable was included as a confounder, this erroneous omission can cause a high type I error and wrong estimates. If there is uncertainty about the random‐effect structure or concern about the statistical power, more time should be invested up front in hypothesis design and appropriate power analyses for mixed‐effects models (e.g., Brysbaert & Stevens, 2018; Green & MacLeod, 2016).

## 5 CONCLUSION

In conclusion, we showed that mixed‐effects models are more robust than previously thought, despite the biased variance estimates for low number of levels in the grouping variable. We found that power and type I error of the population‐level effect are robust against the model choice when the ecological effect is the same among the levels of the grouping variable, however, the model matters when the ecological effect differs among levels. When in doubt about the data‐generating process, we encourage starting with a simplified model (random intercept only) and consult model diagnostics and simulated LRTs to check for evidence of random slope effects. When finding evidence for random slopes in these tests, we recommend starting with the mixed‐effects model and switching only to a fixed‐effects model in case of a singular fit problem. With this work, we provide a practical guideline, which helps analysts in the study design, the data analysis, and thus, making ecological inference more informative and robust.

## AUTHOR CONTRIBUTIONS

**Johannes Oberpriller:** Conceptualization (equal); formal analysis (equal); methodology (equal); software (equal); writing – original draft (equal); writing – review and editing (equal). **Melina de Souza Leite:** Conceptualization (equal); Methodology (equal); writing – original draft (equal); writing – review and editing (equal). **Maximilian Pichler:** Conceptualization (equal); formal analysis (equal); methodology (equal); software (equal); writing – original draft (equal); writing – review and editing (equal).

## CONFLICT OF INTEREST

The authors declare no conflict of interests.

## Supporting information


**Appendix S1** Supporting information.

**Appendix S2** Supplementary material.

## Data Availability

No empirical data was used in this study. Code to run and analyze the experiments can be found at https://zenodo.org/record/5817298#.YdRJ9VMxnRY.
