# Supplementary material for: Fixed or random? On the reliability of mixed‐effects models for a small number of levels in grouping variables
Source: Ecol Evol. 2022 Jul 24;12(7):e9062. doi: 10.1002/ece3.9062 (PMC9309037; doi:10.1002/ece3.9062)
Supplement: Supplementary file 1 — Appendix S1 Supporting information. [file ECE3-12-e9062-s002.docx]

# Supporting Information S1 - Fixed or random? On the reliability of mixed-effect models for a small number of levels in grouping variables

## Additional Results for linear-mixed effect models

### Results for the intercept

We calculated type I error rate and power for the intercept estimates of Scenario A and B (Fig. S1, Fig. S2) and we found similar patterns as for the slope estimates (Fig. 1, Fig. 2).


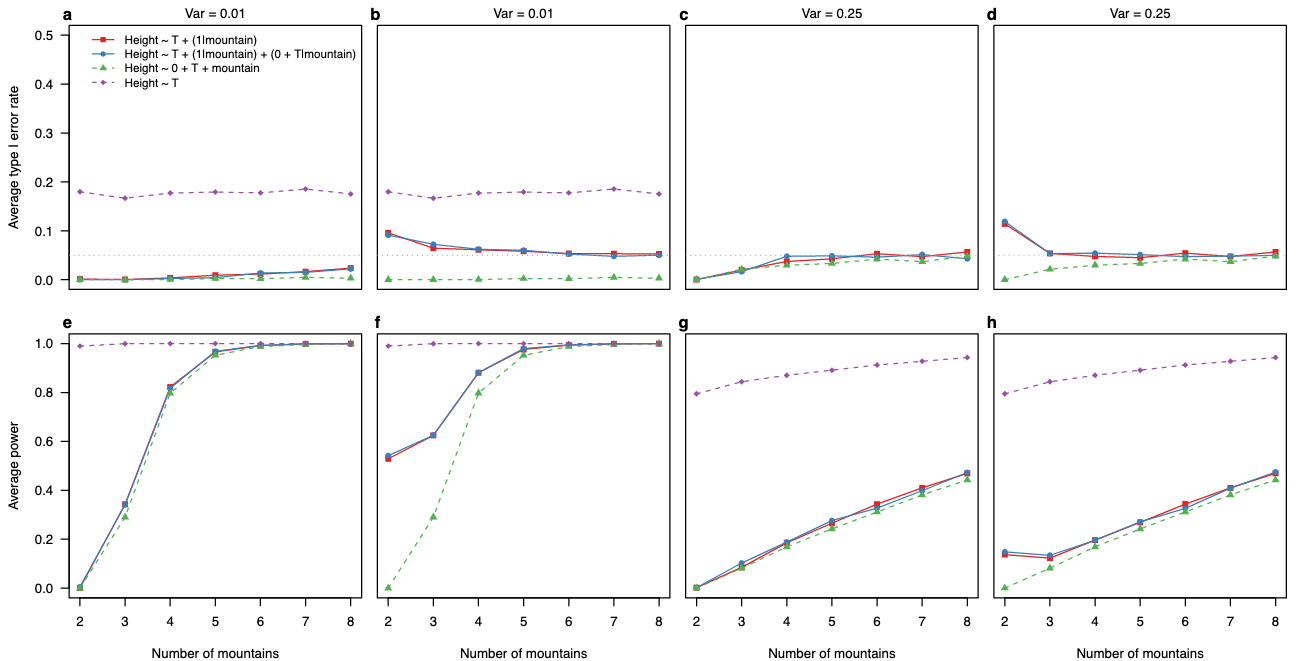


**Figure S1**: Average type I error rates and average power for the intercept in linear fixed and mixed-effects models fitted to simulated data with 2-8 mountains (random intercept for each mountain - Scenario A) and with 50 observations per mountain. For each scenario, 5000 simulations and models were tested. (a, b, e, f) show results for simulated data with a variance of 0.01 in the random effects. (c, d, g, h) show results for simulated data with a variance of 0.25 in the random effects. (a, c, e, g) show results for mixed-effects models only from datasets in which mixed-effects models converged without presenting singular fit problems and (b, d, f, h) results for mixed-effects models for all datasets. Results for fixed-effects (a-h) model are from all datasets. (a-d) the dotted line represents the 5% alpha level.


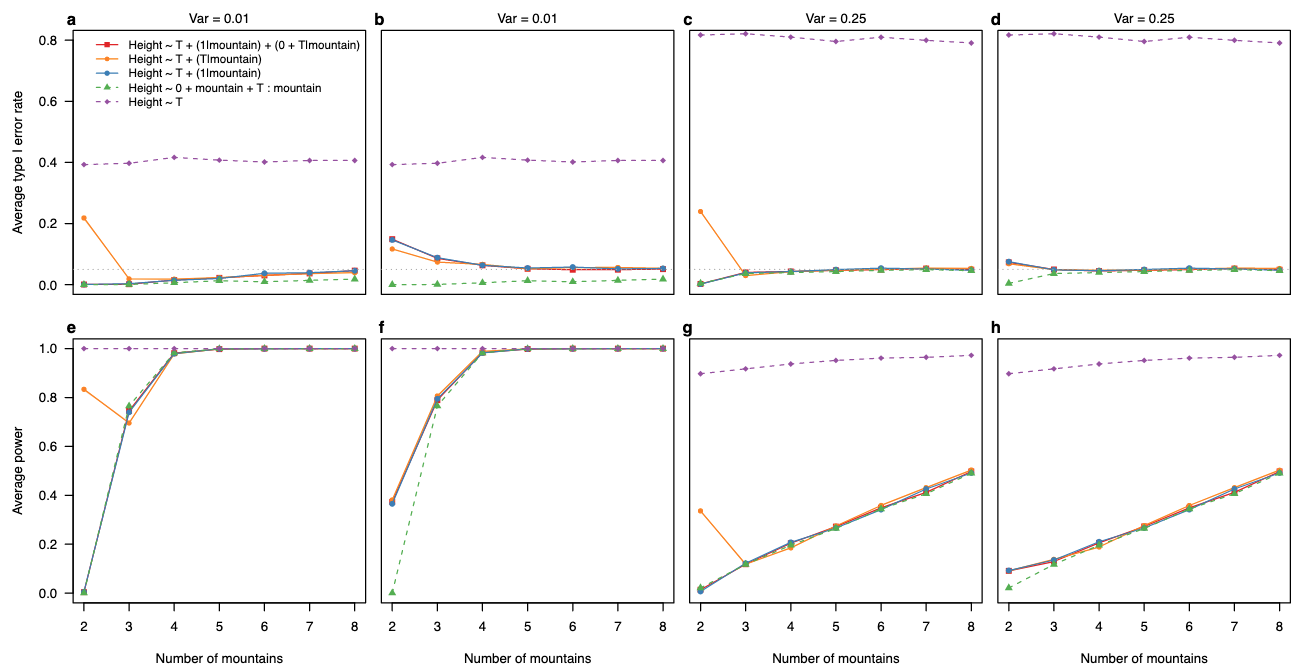


**Figure S2**: Average type I error rates and average power for the intercept in linear (mixed-effects) models fitted to simulated data with 2-8 mountains for scenario B (random intercept and random slope for each mountain range). For each scenario, 5.000 simulations and models were tested. (a, b, e, f) show results for simulated data with a variance of 0.01 in the random effects. (c, d, g, h) show results for simulated data with a variance of 0.25 in the random effects. (a, c, e, g) show results for mixed-effects models only from datasets in which mixed-effects models converged without presenting singular fit problems and (b, d, f, h) results for mixed-effects models for all datasets. Results for fixed-effects (a-h) model are from all datasets. In (a-d) the dotted line represents the 5% alpha level.

### Variance estimates and singular fits

We found that singular fits occurred more often in mixed-effect models when using MLE compared to REML (Table S1). The rate of singular fits decreased with increasing number of groups (Table S1).

Additional to the rate of singular fits a direct comparison of the variance estimates using REML and MLE is necessary to compare their performance. Using MLE for linear mixed-effect models led to stronger towards zero biased estimates compared to using REML (Fig. S3). Estimates for balanced and unbalanced data do not differ within REML and MLE (Fig. S4, S5)

**Table S1:** Rate of models with singular fit convergence problem in *lme4* when using maximum likelihood (MLE) and restricted maximum likelihood (REML) fitting algorithms. Notice that for GLMMs in *lme4*, REML is not implemented.

|  | **LMM** | | **GLMM** |
| --- | --- | --- | --- |
| **Number of groups** | **REML** | **MLE** | **MLE** |
| 2 | 77% | 92% | 95% |
| 3 | 64% | 80% | 89% |
| 4 | 55% | 70% | 84% |
| 5 | 45% | 61% | 81% |
| 6 | 40% | 54% | 78% |
| 7 | 36% | 48% | 76% |
| 8 | 32% | 43% | 72% |


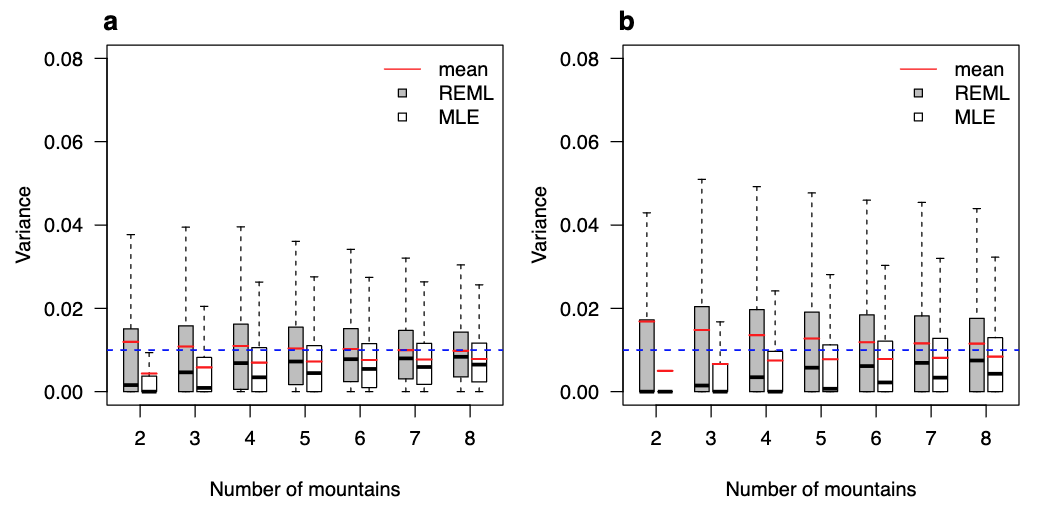


**Figure S3**: Variance estimates of the random intercepts (a) and random slopes (b) for **linear mixed-effect models** (LMM) fitted to simulated data with 2-8 numbers of artificial mountain ranges. For each scenario, 5,000 simulations and models were tested. The blue line represents the true variance used in the simulation (0.01). The grey boxes show results for the models fitted by restricted maximum likelihood estimation (REML) and the white boxes shows results for the models fitted by maximum likelihood estimation (MLE).


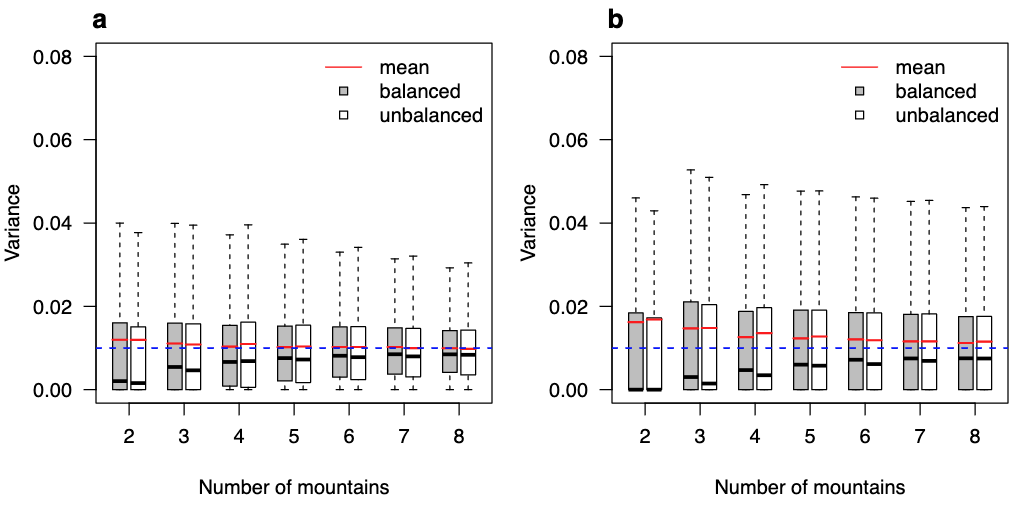


**Figure S4**: Variance estimates of the random intercepts (a) and random slopes (b) for **linear mixed-effect models** (LMM) fitted to simulated data with 2-8 numbers of artificial mountain ranges using REML. For each scenario, 5,000 simulations and models were tested. The blue line represents the true variance used in the simulation (0.01). The grey boxes show the results for the models with unbalanced data (number of observation) among mountains and the white boxes shows results for the models fitted with balanced data among mountains.


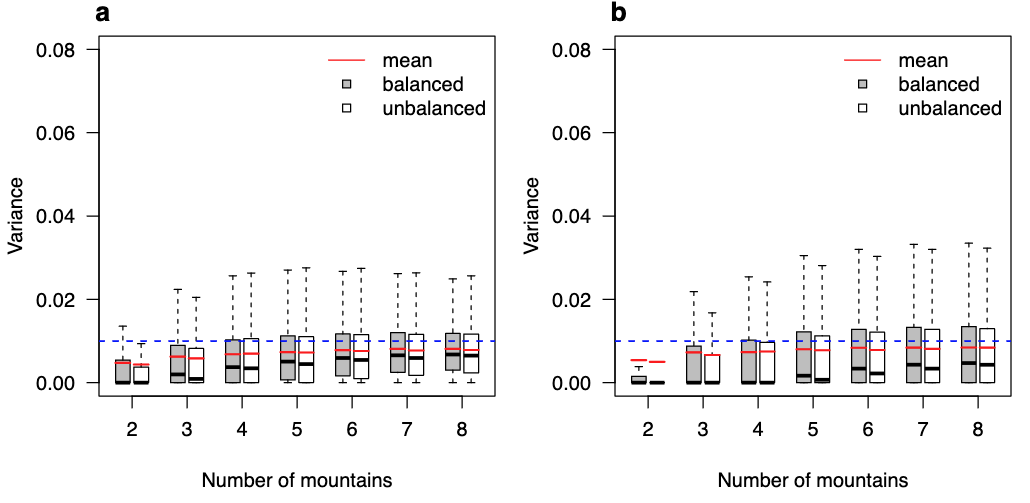


**Figure S5**: Variance estimates of the random intercepts (a) and random slopes (b) for **linear mixed-effect models** (LMM) fitted to simulated data with 2-8 numbers of artificial mountain ranges using MLE. For each scenario, 5,000 simulations and models were tested. The blue line represents the true variance used in the simulation (0.01). The grey boxes show the results for the models with unbalanced data (number of observation) among mountains and the white boxes shows results for the models fitted with balanced data among mountains.

## Results for generalized linear-mixed effect models

If we would have also hypothesized that higher temperatures increase the reproductive success (either yes or no) of a plant species (H2), we would have to test also generalized mixed-effect models. To do so, we also simulated an unbalanced study design for this hypothesis with 2-8 mountains from a varying number of plants for each mountain (H2: expected range between 40-360 plants per mountain) while keeping the overall number of plants constant along altitudinal transects.

Again, we simulated 5000 datasets for each case. The used the inverse logit link function and sampled from a binomial distribution. We used models GLMs and GLMMs to fit the models to the simulated data with binominal distribution.

We found similar patterns as for the LM and LMMs in the main text. For scenario A, the number of mountains didn’t affect the type I error rates or the statistical power, regardless of if the singular fits were included in the results for the mixed-effect models or not (Fig. S6). Here, we didn’t test the overparametrized model or higher variances (larger effect sizes) in the simulated random intercepts.

For scenario B, the overall pattern was again very similar to the findings for the LM and LMMs (Fig. 2). Type I error rates were robust to the number of the levels in the grouping variable (Fig. S7a-d), but the rates for all models were affected by the variance of the random effects (the size of random effects), and the mixed-effect models showed increased type I error rates when the singular fits were included (Fig. S7b, d).

The fixed-effect model without the grouping variable showed for low variance in the random effects close to the nominal level average type I error rates. However, for high variances, the average type I error rates were highly increased.

One notable difference to LM and LMMs is that the overall power of the mixed-effect models was always higher than when modeling the grouping variable as a fixed effect (Fig. S7e-h). This difference in power was here higher than for LMs and LMMs (Fig. 2).


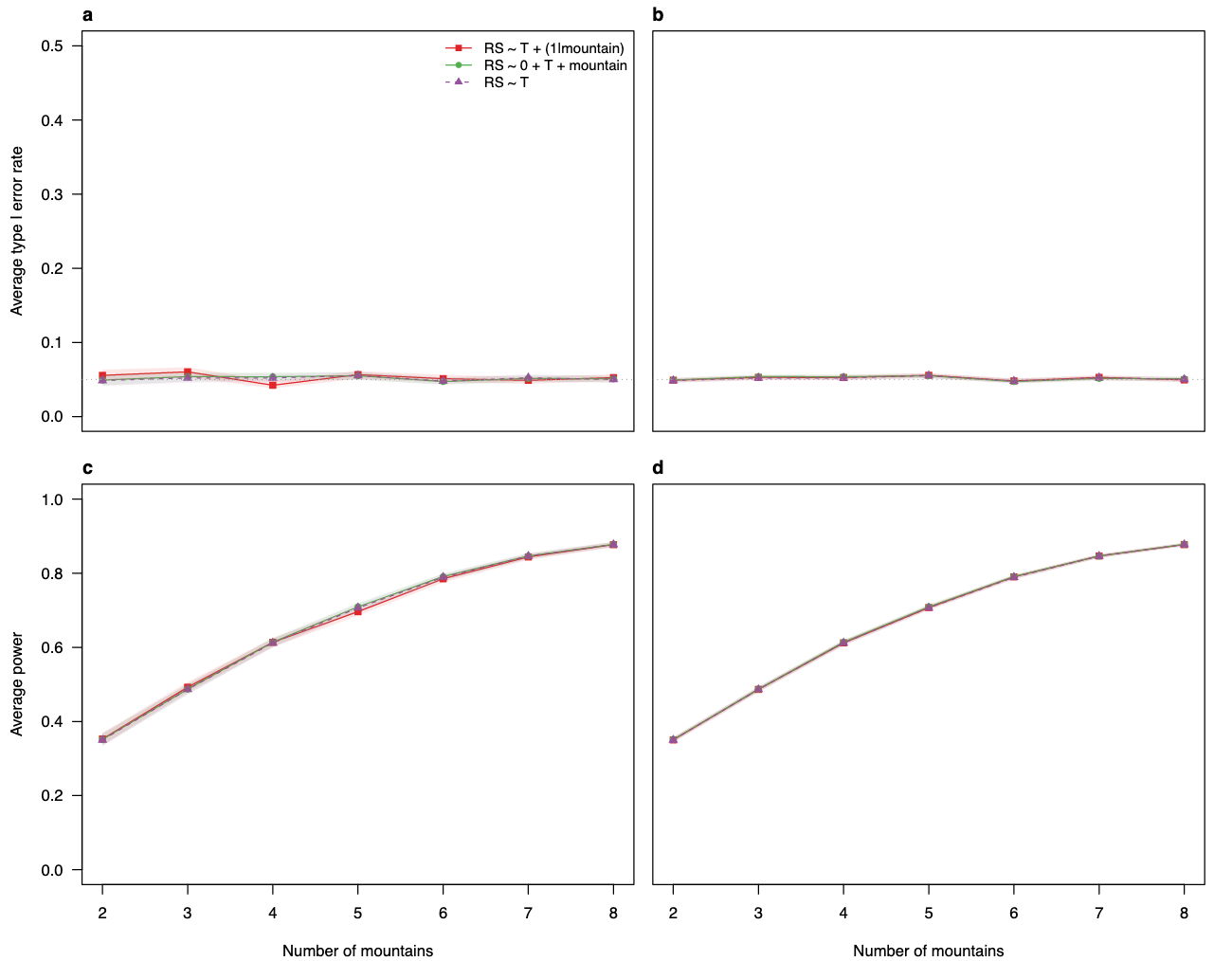


**Figure S6**: Average type I error rates and average power for generalized linear fixed and mixed-effect models fitted to simulated data with 2-8 mountains (random intercept for each mountain - Scenario A) and with 200 observations per mountain. For each scenario, 5000 simulations and models were tested. (a, c) show results for mixed-effects models only from datasets in which mixed-effects models converged without presenting singular fit problems and (b, d) results for mixed-effects models for all datasets. Results for fixed-effects (a-d) model are from all datasets. (a-d) the dotted line represents the 5% alpha level.


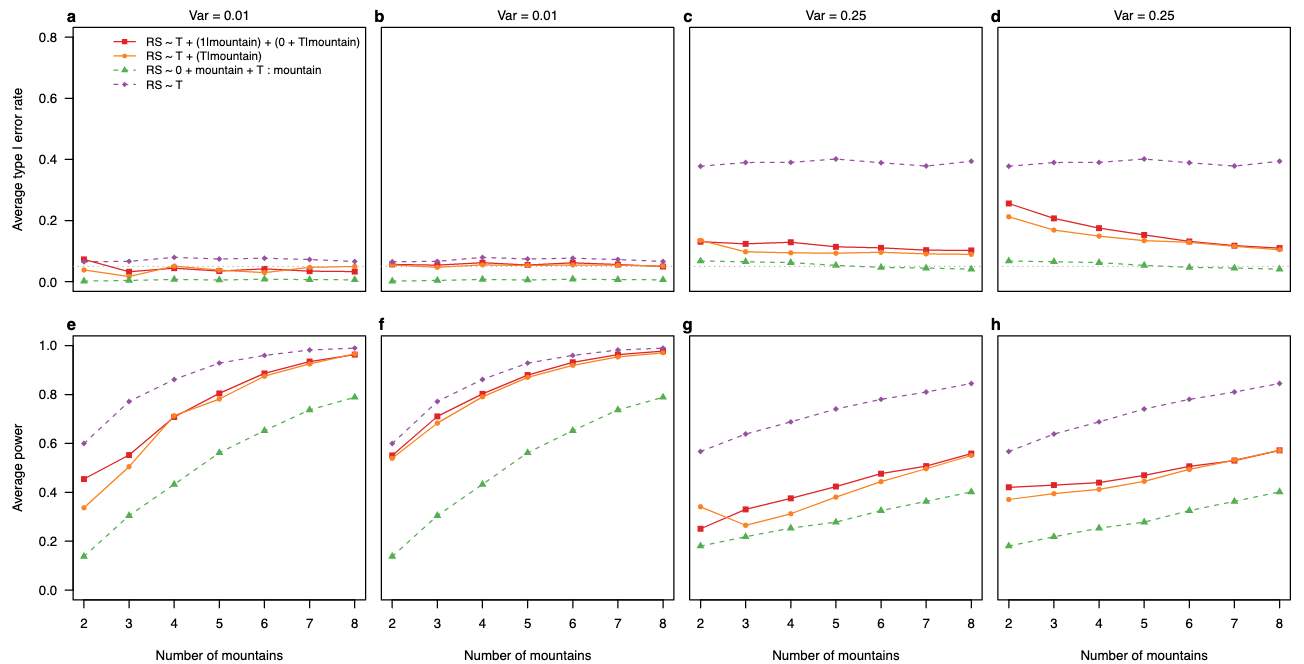


**Figure S7**: Average type I error rates and average power for generalized linear (mixed-effect) models fitted to simulated data with 2-8 mountains for scenario B (random intercept and random slope for each mountain range). For each scenario, 5.000 simulations and models were tested. (a, b, e, f) show results for simulated data with a variance of 0.01 in the random effects. (c, d, g, h) show results for simulated data with a variance of 0.25 in the random effects. (a, c, e, g) show results for mixed-effects models only from datasets in which mixed-effects models converged without presenting singular fit problems and (b, d, f, h) results for mixed-effects models for all datasets. Results for fixed-effects (a-h) model are from all datasets. In (a-d) the dotted line represents the 5% alpha level.

We calculated the statistical properties for the intercept estimates of Scenario A and B (Fig. S8, Fig. S9) and we found similar patterns as for the slope estimates (Fig. 1, Fig. 2).

Also, the distribution of the variance estimates of the random effects matched with the results of the LMMs (Fig. 3, Fig. S10)


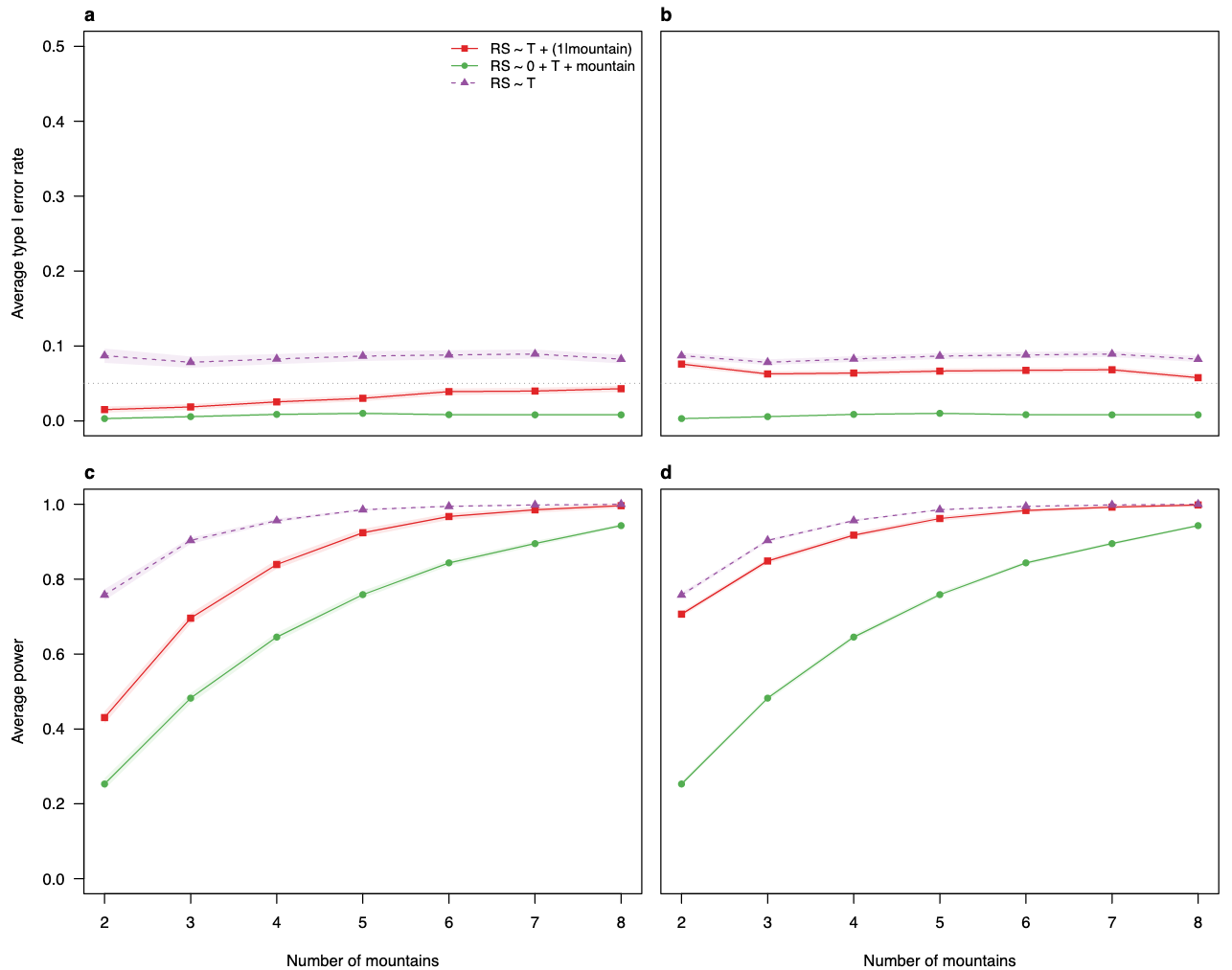


**Figure S8**: Average type I error rates and average power for the intercept in generalized linear fixed and mixed-effect models fitted to simulated data with 2-8 mountains (random intercept for each mountain - Scenario A) and with 200 observations per mountain. For each scenario, 5000 simulations and models were tested. (a, c) show results for mixed-effects models only from datasets in which mixed-effects models converged without presenting singular fit problems and (b, d) results for mixed-effects models for all datasets. Results for fixed-effects (a-d) model are from all datasets. (a-d) the dotted line represents the 5% alpha level.


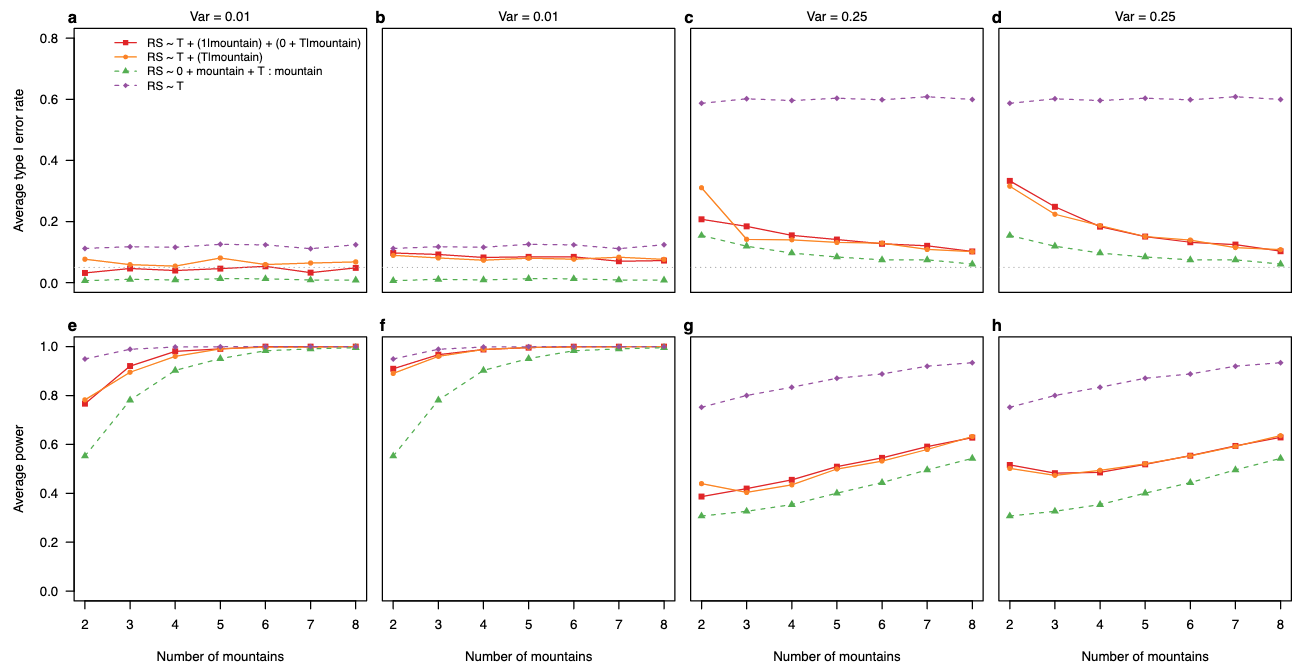


**Figure S9**: Average type I error rates and average power for the intercept in generalized linear (mixed-effect) models fitted to simulated data with 2-8 mountains for scenario B (random intercept and random slope for each mountain range). For each scenario, 5.000 simulations and models were tested. (a, b, e, f) show results for simulated data with a variance of 0.01 in the random effects. (c, d, g, h) show results for simulated data with a variance of 0.25 in the random effects. (a, c, e, g) show results for mixed-effects models only from datasets in which mixed-effects models converged without presenting singular fit problems and (b, d, f, h) results for mixed-effects models for all datasets. Results for fixed-effects (a-h) model are from all datasets. In (a-d) the dotted line represents the 5% alpha level.


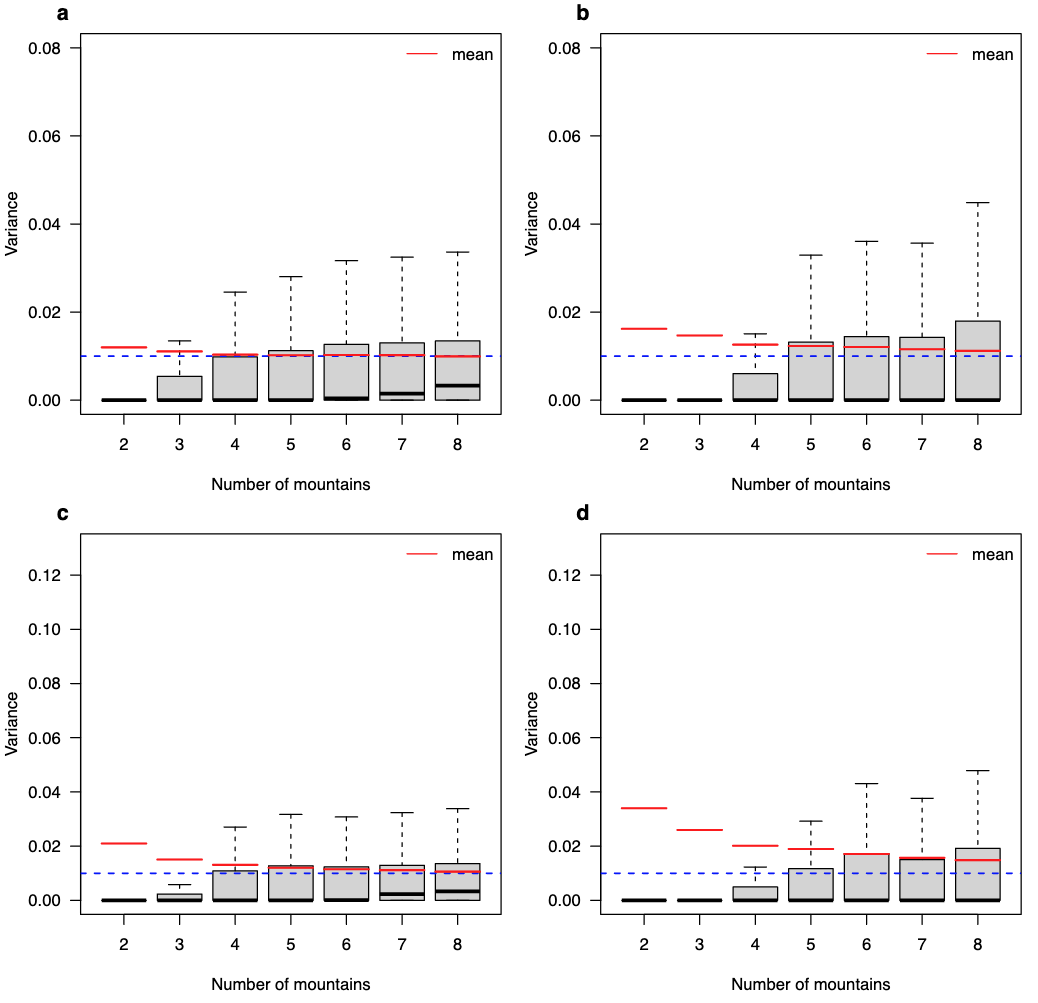


**Figure S10**: Variance estimates of random intercepts (a, c) and random slopes (b, d) for generalized linear mixed-effects models in Scenario B, fitted with *lme4* using MLE to simulated data with 2-8 mountains. Figures (a) and (b) show the results for all models (singular and non-singular fits) and figures (c) and (d) show the results for only non-singular fits. For each scenario, 5.000 simulations and models were tested. The blue dotted lines represent the true variance used in the simulation (0.01) and the red lines the average variance estimates.

## Calculation of the mean temperature effect in fixed-effect models with interaction

As fixed-effect models with interactions estimate the effect of one level and its contrasts to the other levels, the population effect of temperature itself is not estimated in the R default parametrization. To calculate the population effect and its significance to be able to compare it to mixed-effect models result, we estimate the grand mean and its standard error via bootstrapping. To do so, we sample from the multivariate normal distribution reported from the fitted linear for the interactions. Let S be a big enough sample from this distribution, then the mean of S is the grand mean temperature effect, and the standard error of S is the standard error of the mean. In R language we can calculate p-values using 2000 samples given the effect estimates *effects* and the covariance matrix of the individual level effects *V* from the linear model in the following way:

| S = mvtnorm::rmvnorm(2000, mean = effects, sigma = V) |
| --- |
|  |

| eff = mean(S) |
| --- |
|  |

| se = sd(S)/sqrt(mountain-1) |
| --- |
|  |

|  |
| --- |
|  |

|  |
| --- |

We then used t-values for LMs and z-values for GLMs for a better comparison with the respective packages to calculate p-values.

To check whether our calculation of the grand mean behaves approximatively correctly, i.e. with no effect we would expect a uniform distribution of the p-values, we simulated scenario B with no grand mean (effect was set to 0), different standard deviations of the varying random intercept and slope (0.1, 0.5, and 1.0), and unbalanced (30 observations for each group but 1000 observations for the last one) and balanced (30 observations for each group) study design. We found that the distribution of p-values is approximatively correct for larger standard deviations (which is linked to the number of observations in each group, Fig. S11).

We repeated the simulations for GLMs and we found that the distributions of p-values were on average left-skewed (Fig. S12). The nature of the logit link in the GLM explains the increase in left skewness with higher standard deviations of the random effects (Fig. S12).

Using zero sum contrasts is another way to calculate the average affects over a grouping variable. We repeated the above-mentioned simulation to compare the zero-sum contrasts method to our bootstrapping approach. However, we found highly right-skewed distribution of p-values for the zero-sum contrasts for both models, the LM and the GLM (Fig. S13, S14). Thus, the zero-sum contrasts method leads to high type I errors and very high power for LMs and GLMs, while our bootstrapping method leads to correct type I errors and power for LMs, but higher than expected type I error rates and less power for the GLMs. Therefore, we think that our bootstrapping approach is preferable over the zero-sum contrast method.


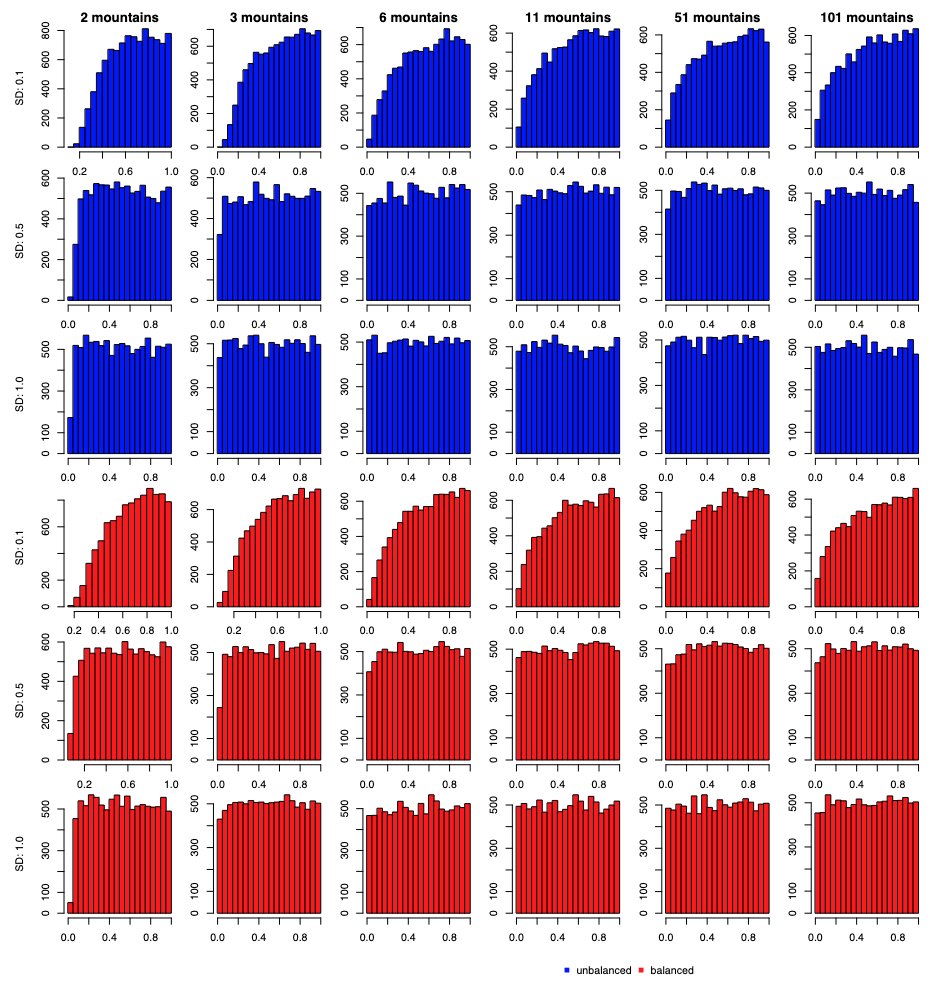


**Figure S11:** The distribution of p-values (based on t-statistics) for the grand mean calculation of linear models for different simulation scenarios: 2 – 101 mountains, different SDs for the random effects (0.1, 0.5, and 1.0), and for unbalanced (blue) and balanced (red) study design. Each scenario was simulated 10,000 times.

**
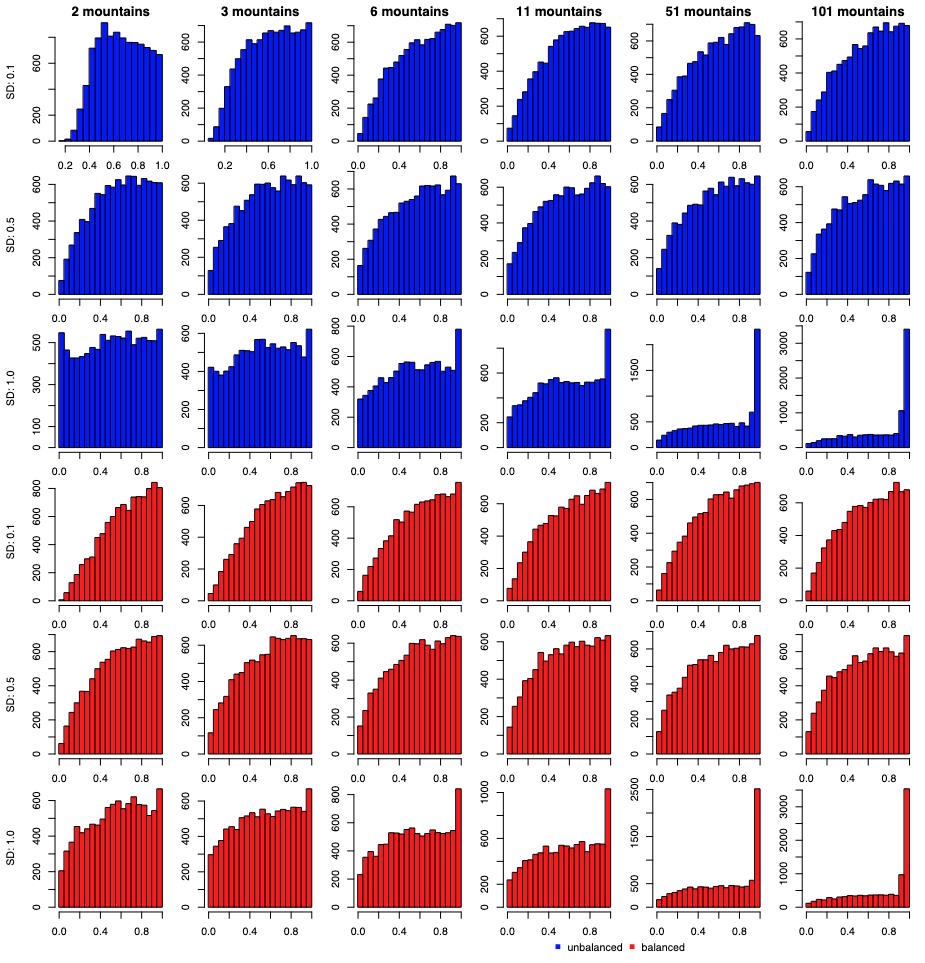
**

**Figure S12:** The distribution of p-values (based on z-statistics) for the grand mean calculation of generalized linear models for different simulation scenarios: 2 – 101 mountains, different SDs for the random effects (0.1, 0.5, and 1.0), and for unbalanced (blue) and balanced (red) study design. Each scenario was simulated 10,000 times.


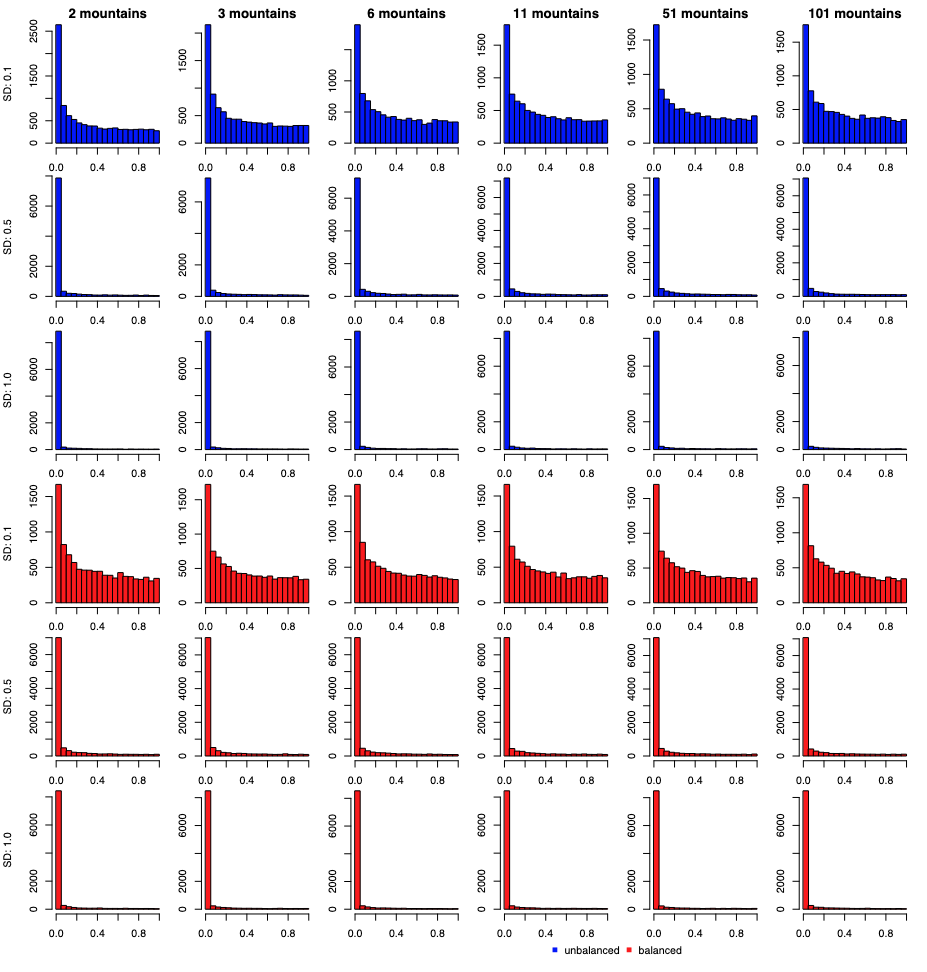


**Figure S13:** The distribution of p-values (based on t-statistics) for the grand mean calculation in linear models (based on zero sum contrasts) for different simulation scenarios: 2 – 101 mountains, different SDs for the random effects (0.1, 0.5, and 1.0), and for unbalanced (blue) and balanced (red) study design. Each scenario was simulated 10,000 times.


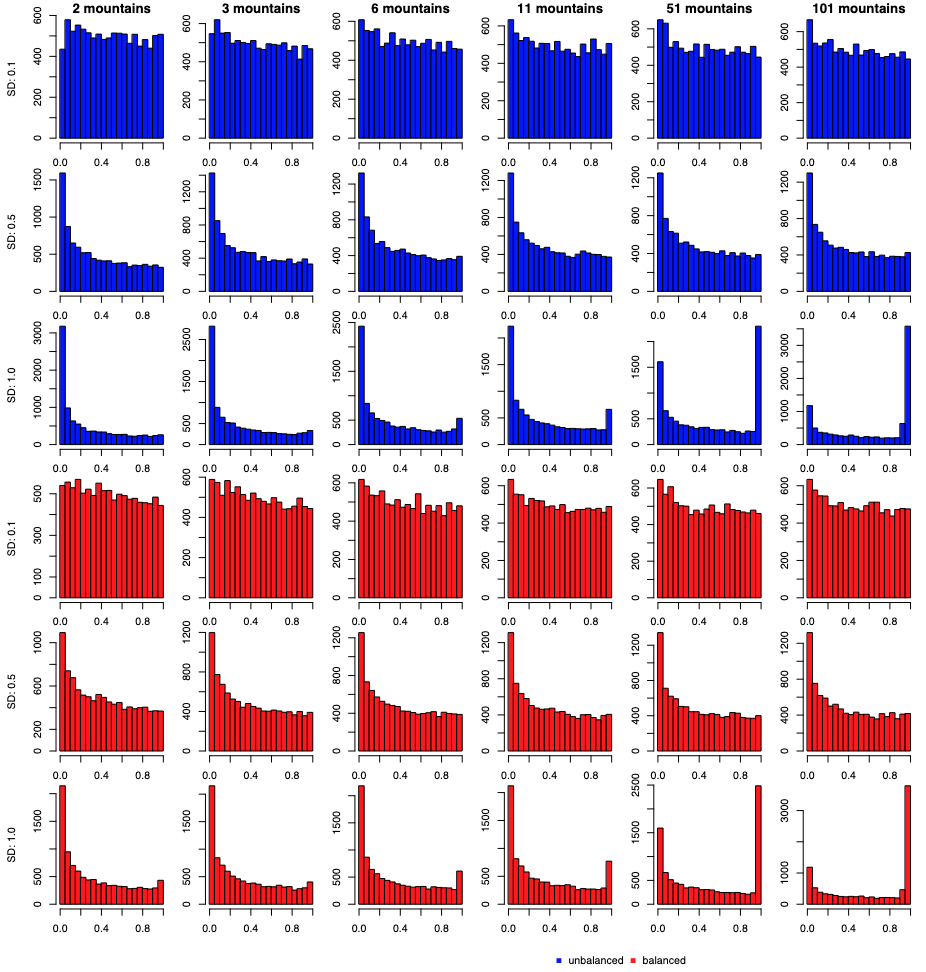


**Figure S14:** The distribution of p-values (based on t-statistics) for the grand mean calculation in generalized models (based on zero sum contrasts) for different simulation scenarios: 2 – 101 mountains, different SDs for the random effects (0.1, 0.5, and 1.0), and for unbalanced (blue) and balanced (red) study design. Each scenario was simulated 10,000 times.

### Distribution of population-level effect estimates

To check what causes different type I error rates, we plotted the distribution of estimated population mean effects for scenario B for a different number of mountains (2, 3, 5, and 7, corresponding to panels a-d in Fig. S21 and S22) for the intercept (Fig. S21) as well as for the slope (Fig. S22). We found no differences for the different models for the estimates of the population effect (Fig. S21, 22). We found that with increasing number of mountains the variance of the distribution declines.


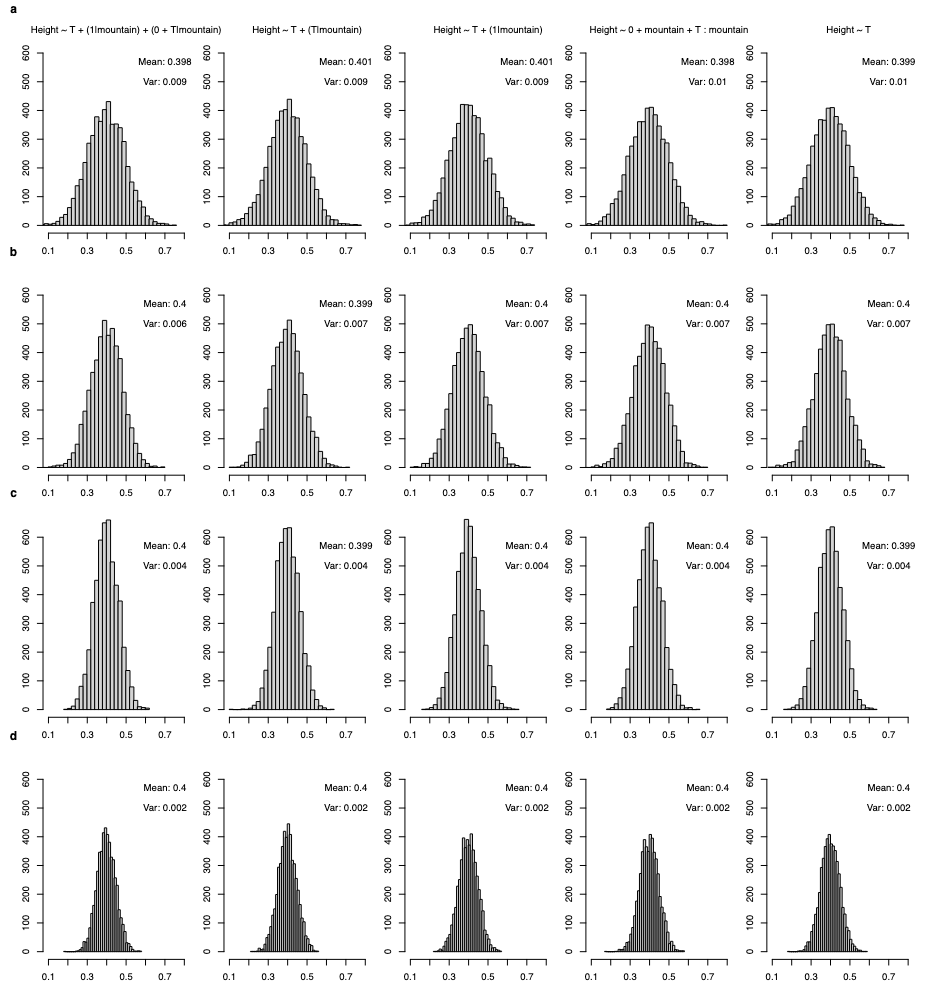


**Figure S15:** The distribution of the population slope effect (grand mean, ecological effect) of temperature for scenario B. a-d show different number of mountains (2, 3, 5, and 7) and each column corresponds for a different model which we tested for scenario B (see Figure 2 in main text).


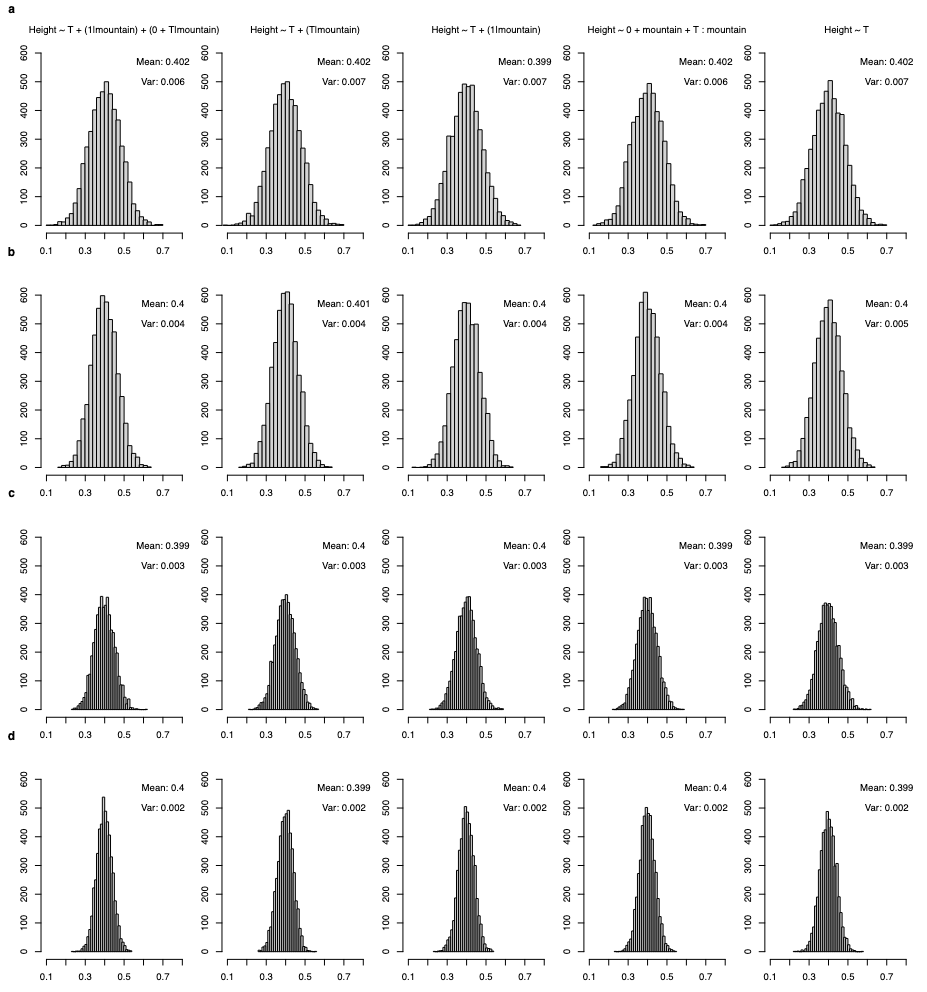


**Figure S16:** The distribution of the population intercept effect for scenario B. a-d show different number of mountains (2, 3, 5, and 7) and each column corresponds for a different model which we tested for scenario B (see Figure 2 in main text).

## References

Bates, D., Mächler, M., Bolker, B., & Walker, S. (2015). Fitting Linear Mixed-Effects Models Using lme4. *Journal of Statistical Software*, *67*(1), 1–48. https://doi.org/10.18637/jss.v067.i01

Brooks, M. E., Kristensen, K., Benthem, K. J. van, Magnusson, A., Berg, C. W., Nielsen, A., Skaug, H. J., Maechler, M., & Bolker, B. M. (2017). Modeling Zero-Inflated Count Data With glmmTMB. *BioRxiv*, 132753. https://doi.org/10.1101/132753

Kristensen, K., Nielsen, A., Berg, C. W., Skaug, H., & Bell, B. M. (2016). TMB: Automatic Differentiation and Laplace Approximation. *Journal of Statistical Software*, *70*(5), 1–21. https://doi.org/10.18637/jss.v070.i05
