# Supplementary material for: Fixed or random? On the reliability of mixed‐effects models for a small number of levels in grouping variables
Source: Ecol Evol. 2022 Jul 24;12(7):e9062. doi: 10.1002/ece3.9062 (PMC9309037; doi:10.1002/ece3.9062)
Supplement: Supplementary file 2 — Appendix S2 Supplementary material. [file ECE3-12-e9062-s001.docx]

## Appendix

The R packages *lme4* (Bates et al., 2015) and *glmmTMB* (Brooks et al., 2017) are among the most popular implementations to estimate mixed-effect models. They differ in their optimization routines and the calculation of p-values for linear mixed-effect models (LMMs). *glmmTMB* can fit linear and generalized linear models using maximum likelihood (MLE) and the restricted maximum likelihood estimation (REML) whereas *lme4* only supports REML for LMMs but not for GLMMs. By default, *lme4* uses REML for LMMs (*lmer* function) and MLE for GLMMs (*glmer* function), while *glmmTMB* uses MLE by default for any kind of data.

We repeated the experiments describe above with *glmmTMB* instead of *lme4*. One striking difference is that *glmmTMB* doesn’t report singular fits. This not attributable to a technical difference, but rather *lme4* defines a singular fit when the estimated variance of a random effect is smaller than 10^-4^. Thus, we decided to use the same threshold for *glmmTMB* (which has no default threshold) to classify variance estimates as singular fits or not.

### Statistical properties using *glmmTMB*

For scenario A, the patterns we found for average type I error rate and average statistical power of the population-level effect (effect size of the temperature predictor) were similar to the findings of *lme4* (Fig. 1, Fig. A1). However, the overparametrized model was less affected by the variance of the random effects or the consideration of singular fits. The average power of the overparametrized model was higher than when using *lme4.*

For scenario B, again the patterns were similar to *lme4* with the exception that for higher variances, the average type I error rates of the mixed-effect models estimated by *glmmTMB* were higher than the nominal level, regardless of with or without singular fits. Here, the average type I error rates decreased with the number of the number of mountains to the nominal level (Fig. A2a-d). In *lme4* the average type I error rates were closer to the nominal level (more conservative, Fig. A2). The less conservative average type I error rates led also to higher power (Fig. A2e-h).


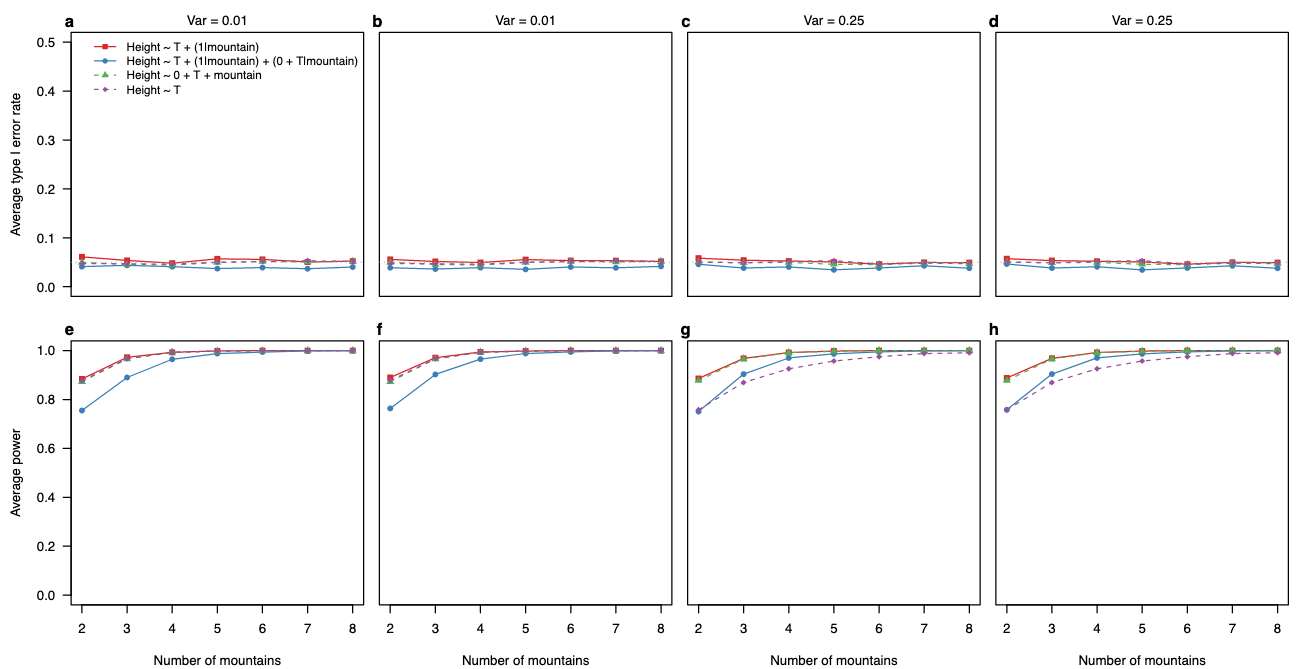


**Figure A1**: Average type I error rates and average power for linear fixed and mixed-effect models (*glmmTMB*) fitted to simulated data with 2-8 mountains (random intercept for each mountain - Scenario A) and with 50 observations per mountain. For each scenario, 5000 simulations and models were tested. (a, b, e, f) show results for simulated data with a variance of 0.01 in the random effects. (c, d, g, h) show results for simulated data with a variance of 0.25 in the random effects. (a, c, e, g) show results for mixed-effects models only from datasets in which mixed-effects models converged without presenting singular fit problems and (b, d, f, h) results for mixed-effects models for all datasets. Results for fixed-effects (a-h) model are from all datasets. (a-d) the dotted line represents the 5% alpha level.


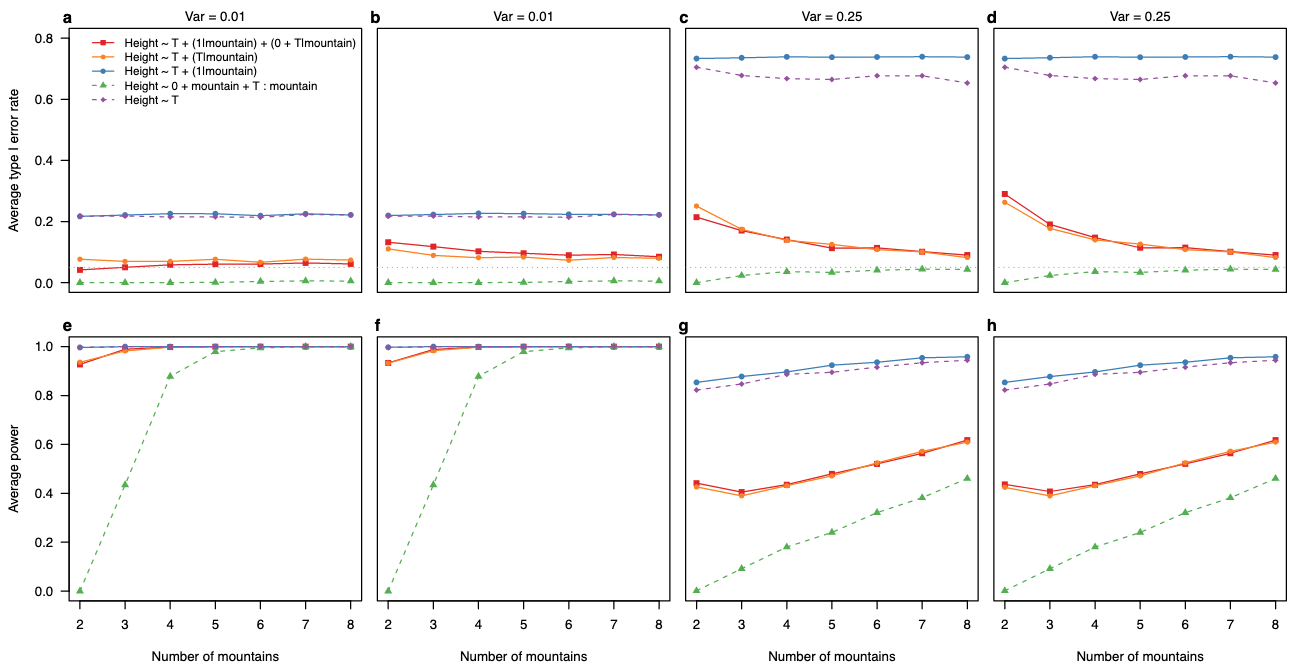


**Figure A2**: Average type I error rates and average power for linear (mixed-effect) models fitted to simulated data with 2-8 mountains for scenario B (random intercept and random slope for each mountain range) using glmmTMB. For each scenario, 5.000 simulations and models were tested. (a, b, e, f) show results for simulated data with a variance of 0.01 in the random effects. (c, d, g, h) show results for simulated data with a variance of 0.25 in the random effects. (a, c, e, g) show results for mixed-effects models only from datasets in which mixed-effects models converged without presenting singular fit problems and (b, d, f, h) results for mixed-effects models for all datasets. Results for fixed-effects (a-h) model are from all datasets. In (a-d) the dotted line represents the 5% alpha level.

For the intercept, we found a similar pattern as for the slopes: The average type I error rates of the mixed-effect models fitted by *glmmTMB* were on average higher for low number of levels and stronger affected by larger random effect sizes than when fitted by *lme4* (Fig. A3, A4, compare with Supplementary Information S1).


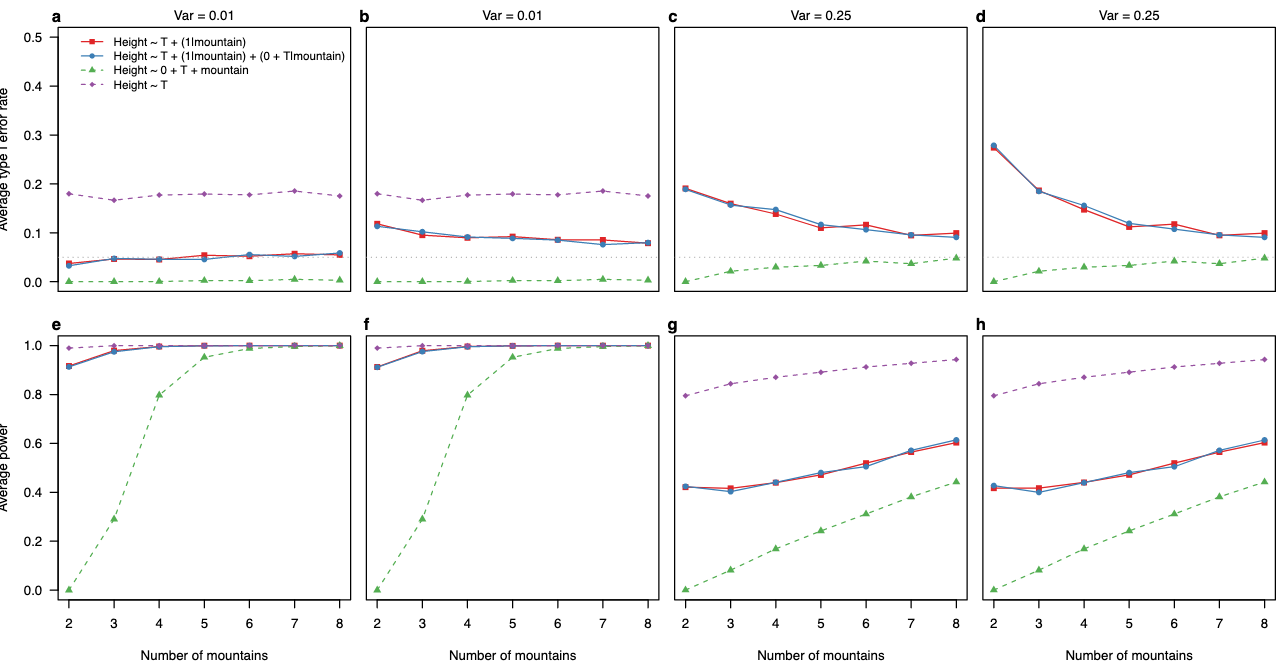


**Figure A3**: Average type I error rates and average power for the intercept in linear fixed and mixed-effect models (*glmmTMB*) fitted to simulated data with 2-8 mountains (random intercept for each mountain - Scenario A) and with 50 observations per mountain. For each scenario, 5000 simulations and models were tested. (a, b, e, f) show results for simulated data with a variance of 0.01 in the random effects. (c, d, g, h) show results for simulated data with a variance of 0.25 in the random effects. (a, c, e, g) show results for mixed-effects models only from datasets in which mixed-effects models converged without presenting singular fit problems and (b, d, f, h) results for mixed-effects models for all datasets. Results for fixed-effects (a-h) model are from all datasets. (a-d) the dotted line represents the 5% alpha level.


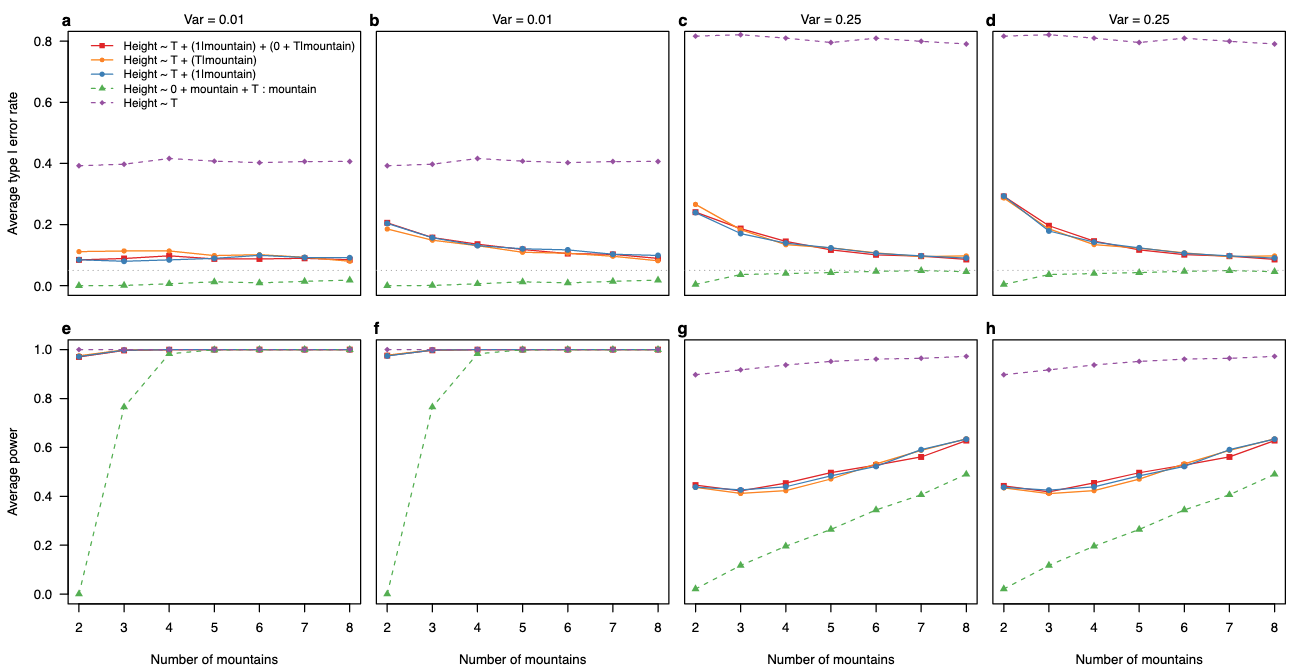


**Figure A4**: Average type I error rates and average power for the intercept in linear mixed-effect models (*glmmTMB*) fitted to simulated data with 2-8 mountains for scenario B (random intercept and random slope for each mountain range). For each scenario, 5.000 simulations and models were tested. (a, b, e, f) show results for simulated data with a variance of 0.01 in the random effects. (c, d, g, h) show results for simulated data with a variance of 0.25 in the random effects. (a, c, e, g) show results for mixed-effects models only from datasets in which mixed-effects models converged without presenting singular fit problems and (b, d, f, h) results for mixed-effects models for all datasets. Results for fixed-effects (a-h) model are from all datasets. In (a-d) the dotted line represents the 5% alpha level.

### Variance estimates and singular fits using *glmmTMB*

We found that singular fits occurred more often in mixed-effect models when using MLE compared to REML (Table A1). Also, the rate of singular fits decreased with increasing number of mountains (Table A1). When using a threshold of 10^-4^ (the same as for lme4) to detect singular fits, the rate of singular fits to non-singular fits was the same as for *lme4.*

For non-singular fits, we found that the average variance estimate in the mixed-effect models were closed to the true value of the data generating process than in *lme4* (Fig. 3, Fig. A5). Estimates for balanced and unbalanced data were the same within REML and MLE (Fig. A7, A8).

When directly comparing the variance estimates of REML and MLE, we found that MLE led to stronger zero-biased estimates compared to REML (Fig. A6).


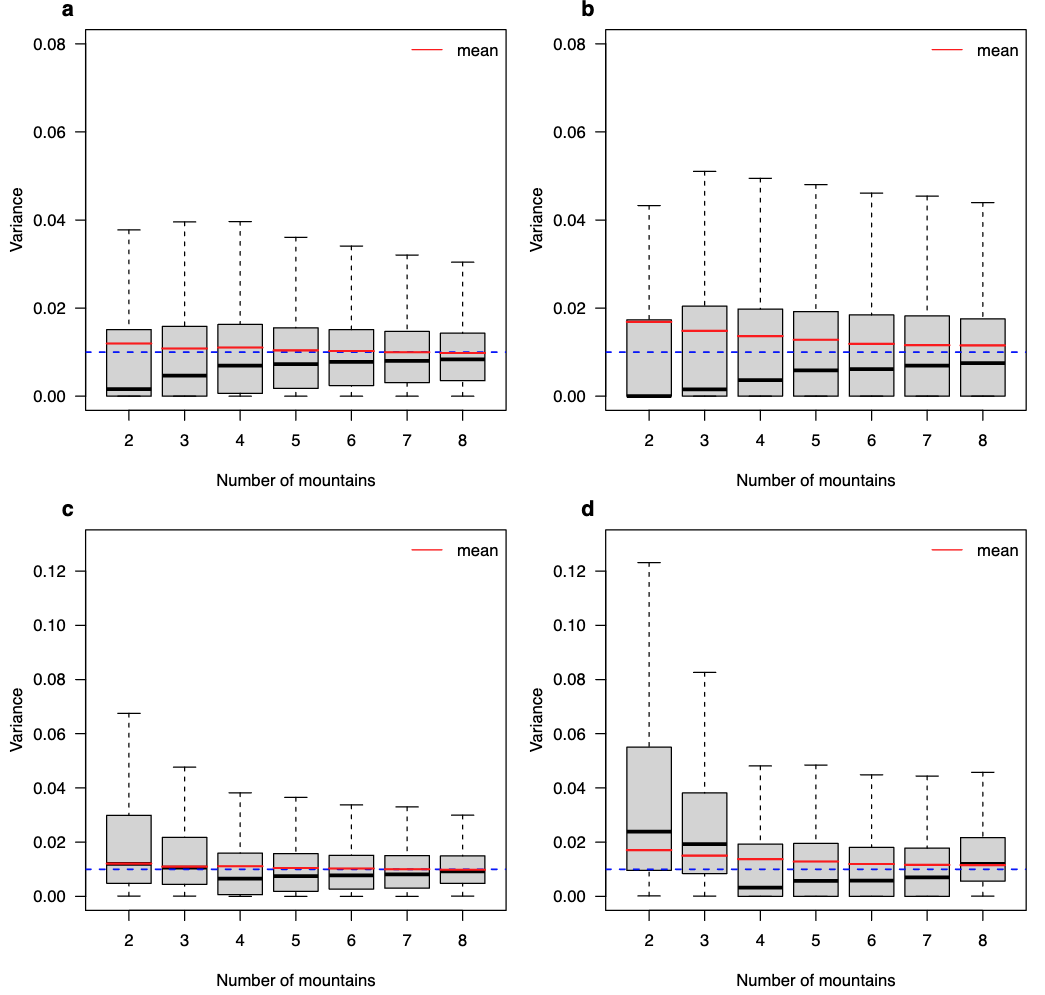


**Figure A5**: Variance estimates of random intercepts (a, c) and random slopes (b, d) for linear mixed-effects models (LMM, Table 1. Eq. M10) in Scenario B, fitted with *glmmTMB* using REML to simulated data with 2-8 mountains. Figures (a) and (b) show the results for all models (singular and non-singular fits) and figures (c) and (d) show the results for only non-singular fits. For each scenario, 5.000 simulations and models were tested. The blue dotted lines represent the true variance used in the simulation (0.01) and the red lines the average variance estimates.

**Table A1:** Proportion of models ran in *glmmTMB* that presented singular fit convergence problem when using maximum likelihood (MLE) and restricted maximum likelihood (REML) fitting algorithms.

|  | **LMM** | | **GLMM** |  |
| --- | --- | --- | --- | --- |
| **Number of groups** | **REML** | **MLE** | **MLE** | **REML** |
| 2 | 77% | 92% | 87% | 97% |
| 3 | 65% | 81% | 80% | 92% |
| 4 | 55% | 71% | 76% | 88% |
| 5 | 46% | 62% | 72% | 84% |
| 6 | 41% | 54% | 69% | 81% |
| 7 | 37% | 48% | 68% | 80% |
| 8 | 33% | 43% | 64% | 76% |


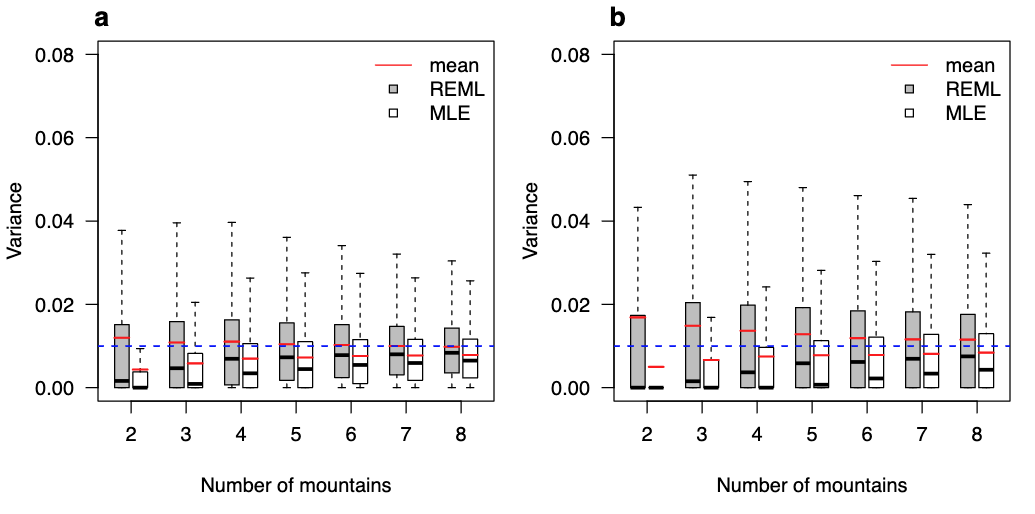


**Figure A6**: Variance estimates of the random intercepts (a) and random slopes (b) for **linear mixed-effect models** (LMM) fitted to simulated data with 2-8 numbers of artificial mountain ranges. For each scenario, 5,000 simulations and models were tested. The blue line represents the true variance used in the simulation (0.01). The grey boxes show results for the models fitted by restricted maximum likelihood estimation (REML) and the white boxes shows results for the models fitted by maximum likelihood estimation (MLE).


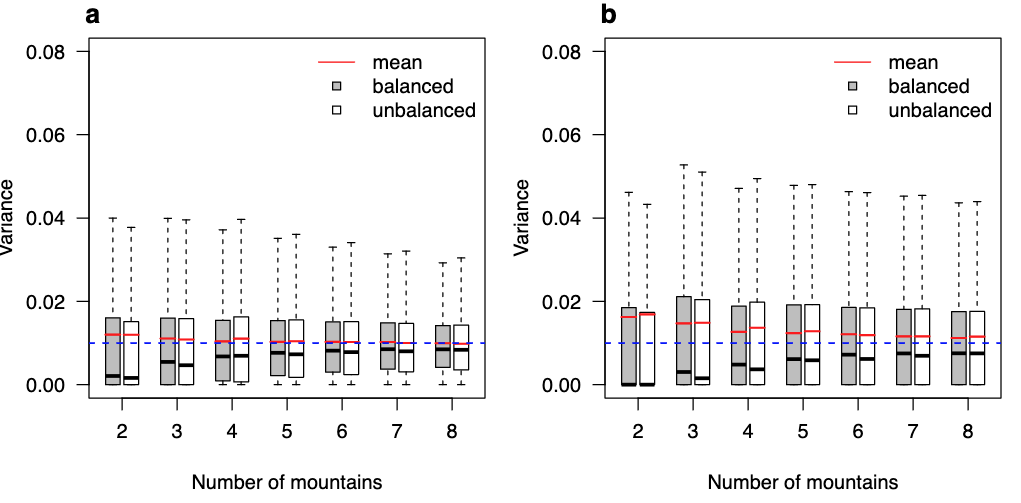


**Figure A7**: Variance estimates of the random intercepts (a) and random slopes (b) for **linear mixed-effect models** (LMM) fitted to simulated data with 2-8 numbers of artificial mountain ranges using REML. For each scenario, 5,000 simulations and models were tested. The blue line represents the true variance used in the simulation (0.01). The grey boxes show the results for the models with unbalanced data (number of observation) among mountains and the white boxes shows results for the models fitted with balanced data among mountains.


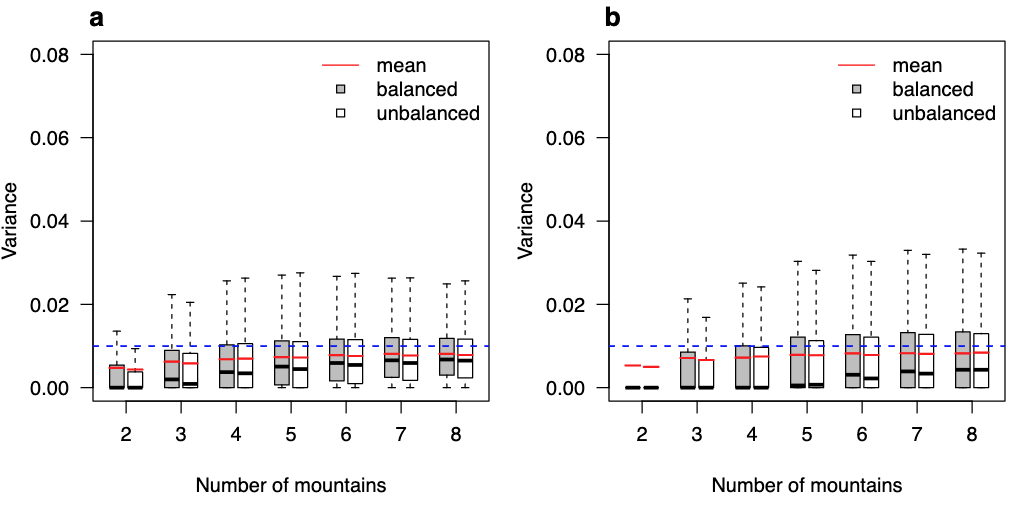
**Figure A8**: Variance estimates of the random intercepts (a) and random slopes (b) for **linear mixed-effect models** (LMM) fitted to simulated data with 2-8 numbers of artificial mountain ranges using MLE. For each scenario, 5,000 simulations and models were tested. The blue line represents the true variance used in the simulation (0.01). The grey boxes show the results for the models with unbalanced data (number of observation) among mountains and the white boxes shows results for the models fitted with balanced data among mountains.

## Summary

At least to some extent, the differences between *glmmTMB* and *lme4* might be explained by the arbitrary chosen threshold to detect singular fits. It is doubtable if the singular fits rate of the two different packages are truly comparable when they are classified with the same threshold but rely on different implementations and optimization routines.

However, even if we include the singular fits in the results of the mixed-effect models, we found that *glmmTMB* showed on average a higher type I error rate for larger variances in the random effect than lme4 (Fig. 1, 2, Fig. A1-A4) indicating that *lme4* can handle in general singular fits better than *glmmTMB* because the average type I error rate of *lme4* was here closer to the nominal level (Fig. 1, 2, Fig. A1-A4). Future work should focus on exploring and understanding the cause of this difference between the two mixed-effect model implementations.

## References Appendix

Bates, D., Mächler, M., Bolker, B., & Walker, S. (2015). Fitting Linear Mixed-Effects Models Using lme4. *Journal of Statistical Software*, *67*(1), 1–48. https://doi.org/10.18637/jss.v067.i01

Brooks, M. E., Kristensen, K., Benthem, K. J. van, Magnusson, A., Berg, C. W., Nielsen, A., Skaug, H. J., Maechler, M., & Bolker, B. M. (2017). Modeling Zero-Inflated Count Data With glmmTMB. *BioRxiv*, 132753. https://doi.org/10.1101/132753

Kristensen, K., Nielsen, A., Berg, C. W., Skaug, H., & Bell, B. M. (2016). TMB: Automatic Differentiation and Laplace Approximation. *Journal of Statistical Software*, *70*(5), 1–21. https://doi.org/10.18637/jss.v070.i05
